# ARIMA-based forecasting of cerebral physiologic signals in acute traumatic brain injury: a CAnadian high-resolution TBI (CAHR–TBI) cohort study

**DOI:** 10.1186/s40635-026-00855-y

**Published:** 2026-03-02

**Authors:** Nuray Vakitbilir, Rahul Raj, Donald E. G. Griesdale, Mypinder Sekhon, Francis Bernard, Clare Gallagher, Eric P. Thelin, Francois Mathieu, Kevin Y. Stein, Andreas H. Kramer, Marcel J. H. Aries, Frederick A. Zeiler

**Affiliations:** 1 Department of Biomedical Engineering, Price Faculty of Engineering, University of Manitoba, Winnipeg, MB Canada; 2 Department of Neurosurgery, University of Helsinki and Helsinki University Hospital, Helsinki, Finland; 3 Department of Anesthesiology, Pharmacology, and Therapeutics, University of British Columbia, Vancouver, BC Canada; 4 Division of Critical Care, Department of Medicine, University of British Columbia, Vancouver, BC Canada; 5 Section of Critical Care, Department of Medicine, University of Montreal, Montreal, QC Canada; 6 Section of Neurosurgery, University of Calgary, Calgary, AB Canada; 7 Department of Clinical Neurosciences, University of Calgary, Calgary, AB Canada; 8 Hotchkiss Brain Institute, University of Calgary, Calgary, AB Canada; 9 Medical Unit Neurology, Karolinska University Hospital, Stockholm, Sweden; 10 Department of Clinical Neuroscience, Karolinska Institutet, Stockholm, Sweden; 11 Division of Neurosurgery, Department of Surgery and Interdepartmental Division of Critical Care, University of Toronto, Toronto, Canada; 12 Department of Critical Care Medicine, University of Calgary, Calgary, AB Canada; 13 Department of Intensive Care, Maastricht University Medical Center+, and School of Mental Health and Neurosciences, University Maastricht, Maastricht, The Netherlands; 14 Section of Neurosurgery, Department of Surgery, Rady Faculty of Health Sciences, University of Manitoba, Winnipeg, MB Canada; 15 Pan Am Clinic Foundation, Winnipeg, MB Canada

**Keywords:** ARIMA, Cerebral physiology, High-frequency signals, Time-series analysis, Multimodal signal analysis

## Abstract

**Background:**

Traumatic brain injury (TBI) remains a major global health issue, with limited progress in reducing morbidity and mortality for TBI patients in need of sedation and intensive care. This has led to increased focus on the mechanisms of secondary brain injury, typically monitored via high-frequency, multi-modal physiologic data reflecting pressure flow and oxygen delivery. However, the complexity and volume of such data pose challenges for clinicians, leading to the use of resolution-reducing techniques, such as moving averages and point sampling. However, data often remains a challenge to utilize clinically for physiologic insult predications and early or pre-emptive interventions. Time series modeling approaches like autoregressive integrated moving average (ARIMA) are valuable in analyzing statistical signal structures, providing insights into temporal dynamics by revealing temporal patterns and forecasting future physiological states.

**Results:**

This study evaluated the effects of resolution reduction via averaging on point and interval predictions using ARIMA models. Analysis was performed on both raw signals and derived physiologic metrics of cerebral pressure flow, compliance, and oxygen delivery by utilizing the CAnadian High-Resolution TBI (CAHR–TBI) data set. Temporal resolution was reduced by averaging with non-overlapping intervals, ranging from 1-min to 24-h windows. Data from A total of 376 TBI patients requiring intensive care was analyzed across various temporal resolutions. ARIMA models perform best at high temporal resolutions, particularly for derived cerebrovascular reactivity indices, with accuracy decreasing for raw signals at lower resolutions. The choice of data partitioning method affects performance; however, all methods struggle at the lowest resolutions, highlighting ARIMA's limitations for long-term forecasting of cerebral physiologic signals with lower resolution data commonly recorded in patient records.

**Conclusions:**

This study highlights the significant influence of temporal resolution and data partitioning methods on the predictive performance of ARIMA models for cerebral physiological signals. While ARIMA performs well at high temporal resolutions, its accuracy declines for raw physiological signals as resolution decreases. The choice of cross-validation method also impacts forecasting performance. The findings underscore the need for hybrid modeling approaches that integrate ARIMA with machine learning techniques to improve predictive accuracy, particularly for complex cerebral physiological signals.

**Supplementary Information:**

The online version contains supplementary material available at 10.1186/s40635-026-00855-y.

## Background

Over the past two decades, traumatic brain injury (TBI) has persisted as a significant global health challenge, with limited advancements in reducing morbidity and mortality in TBI cases requiring intensive care [1]. This lack of advancement has intensified the focus on secondary brain injury mechanisms, particularly cerebral autoregulation and cerebrovascular reactivity (CVR) [2]. Cerebral autoregulation is the brain's ability to maintain stable cerebral blood flow (CBF) despite changes in systemic arterial pressures, a function essential for preventing damage caused by insufficient or excessive blood flow [3]. When autoregulation is impaired, the brain may experience pressure-passive flow states, increasing the risk of hypoperfusion (ischemia) or hyperperfusion (hyperemia), both of which contribute to secondary injury [4]. CVR metrics can be used as proxies for assessing mean arterial pressure (MAP) and intracranial vascular responses [4, 5].

High-frequency signals, such as intracranial pressure (ICP), cerebral perfusion pressure (CPP), and brain tissue oxygenation (PbtO_2_), play a critical role in monitoring cerebral autoregulation and CVR, offering valuable insights into brain function [6–11]. However, the complexity and sheer volume of this data pose significant challenges for real-time analysis, underscoring the importance of developing methods to efficiently process and utilize such information [9, 12]. Similarly, the complexity and volume of high-frequency data pose significant challenges in TBI research, where large data sets are collected over long periods [13–15]. However, even with temporal resolution reduction, we still lack robust methods to manage such data streams for the purposes of predicting or forecasting cerebral physiology. Such forecasting capabilities could prove invaluable for early or pre-emptive interventions aimed at reducing secondary neural injury burden.

To address the challenges posed by high-frequency cerebral physiologic data, time series modeling techniques such as autoregressive integrated moving average (ARIMA) have become instrumental in studying the temporal dynamics of cerebral autoregulation and CVR metrics [16–20]. ARIMA models and forecasts time-dependent data by capturing underlying patterns, such as trends, seasonality, and noise. Its versatility lies in its three components: autoregression (AR), integration (I), and moving average (MA), which allow the model to account for relationships between past and present observations, non-stationarity, and residual errors, respectively [21]. This makes ARIMA particularly suitable for analyzing physiological signals, such as ICP, MAP, and CPP, where trends and variability play crucial roles in understanding pathophysiological processes.

Although more complex forecasting approaches, such as machine learning models, and Kalman-based filters have been applied to biomedical time series, ARIMA was intentionally selected for this study due to its interpretability, computational efficiency, and robustness across varying time-series lengths. These properties make ARIMA particularly suitable for systematically assessing the effects of temporal resolution reduction, as it allows controlled comparisons without the additional data requirements and training complexities associated with nonlinear or deep-learning models. Therefore, ARIMA provides an appropriate baseline model for evaluating how sampling resolution influences forecasting behavior in cerebral physiologic signals.

The temporal resolution of physiologic signals is a key factor influencing the accuracy and interpretability of ARIMA models. High-frequency data often provide detailed insights into short-term dynamics but are computationally expensive and prone to noise, which can obscure meaningful patterns [22]. Temporal resolution reduction, achieved through techniques, such as averaging, offers a practical solution by smoothing high-frequency fluctuations while preserving essential trends. By aggregating data over fixed intervals, e.g., 1-min or 5-min averages, this approach reduces computational complexity and noise, facilitating the application of ARIMA models across diverse resolutions.

Exploring the effects of temporal resolution on ARIMA-based point and interval predictions is critical for optimizing model performance in cerebral physiologic signal analysis. Point prediction focuses on forecasting specific values within a time series, providing direct insights into future signal behavior. In contrast, interval predictions estimate a range of values within which future observations are likely to fall, with a specified level of confidence. These intervals are essential for capturing uncertainty in the predictions, offering a probabilistic framework that enhances reliability.

In this study, the effects of average-sampling resolution reduction on point and interval predictions using the ARIMA model were assessed for various multi-modal cerebral physiologic signals, such as ICP, CPP and PbtO_2_, compliance and CVR metrics. Patient data underwent progressive temporal resolution reduction by averaging within non-overlapping intervals, ranging from 1-min to 24 h. The 1-min resolution represents the highest reliably available temporal resolution in the CAHR–TBI data set after artifact removal and preprocessing, and it also reflects a commonly available export frequency from bedside physiologic monitors, further supporting its relevance for real-world clinical data pipelines. At each resolution, ARIMA analysis was applied to generate point and interval predictions, with performance validated using multiple train/test split methods. This approach aims to systematically evaluate how changes in temporal resolution influence the accuracy of predictions, the reliability of confidence intervals (CI), and the computational feasibility of ARIMA modeling in cerebral physiologic signal analysis.

While prior work has applied ARIMA and related time-series methods to cerebral physiologic signals, existing literature has not examined how progressive temporal resolution reduction affects ARIMA model behavior, forecast accuracy, or confidence interval reliability across multiple cerebral modalities. To our knowledge, this is the first study to provide a systematic, multi-resolution analysis of ARIMA performance across ICP, CPP, PbtO_2_, compliance, and CVR-derived metrics, using both point and interval predictions. By quantifying the trade-offs between temporal resolution, predictive fidelity, and computational efficiency, this work introduces novel insights that can guide the design of future real-time forecasting frameworks and support scalable analytics for high frequency neurocritical care data.

## Materials and methods

### Study participants

This retrospective study utilizes archived data from the CAnadian High-Resolution TBI (CAHR–TBI) Research Collaborative [6], consistent with prior publications from our group [23–28]. The CAHR–TBI data set comprises high-frequency physiologic data prospectively collected from patients aged 18 years or older who were admitted to the intensive care unit (ICU) with moderate-to-severe TBI. Inclusion criteria required a Glasgow Coma Scale (GCS) score below 13, invasive ICP and arterial blood pressure (ABP) monitoring, and data collection initiation within 24 h of hospital presentation. Data were later retrospectively accessed and compiled, with collection periods varying by center: Foothills Medical Centre, University of Calgary (2011–2021); Health Sciences Centre Winnipeg, University of Manitoba (2019–2023); Maastricht University Medical Center, University of Maastricht (2017–2022); and Vancouver General Hospital, University of British Columbia (2014–2019).

### Data collection

High-frequency full waveform physiologic data were recorded in digital time-series format from bedside ICU monitors using the Intensive Care Monitoring "Plus" (ICM +) software (Cambridge Enterprise Ltd, Cambridge, UK; http://icmplus.neurosurg.cam.ac.uk). Data acquisition was performed through either direct digital data transfer or analog-to-digital signal conversion (Data Translations, DT9804 or DT9826). Continuous ABP was measured using a pressure transducer (Edwards, Irvine, CA, USA; Baxter Healthcare Corp. CardioVascular Group, Irvine, CA, USA) zeroed at the level of the tragus via a radial or femoral arterial line.

ICP was monitored using either an intraparenchymal strain gauge probe (Codman ICP MicroSensor, Codman & Shurtlef Inc., Raynham, MA, USA; NEUROVENT–TEMP, RAUMEDIC, Helmbrechts, Germany; Camino ICP Monitor, Natus, Middleton, WI, USA) inserted into the frontal lobe, or an external ventricular drain (EVD; Medtronic, Minneapolis, MN) placed in the lateral ventricle. ICP was acquired at a sampling rate of 100 Hz. Although both EVD and intraparenchymal ICP monitors were present across the cohort, all analyses are performed on a per-patient basis, preventing cross-device mixing and avoiding the need for inter-device calibration adjustments. PbtO_2_ was measured using the Licox Brain Tissue Oxygen Monitoring System (Integra LifeSciences Corp., Plainsboro, NJ) in some patients, at the discretion of the local treatment team.

### Signal processing

The ICM + software was employed for all post-acquisition signal processing. Signal artifacts were removed by qualified personnel blinded to the study objectives and patient information. The cleaning process involved excluding segments with absent or abnormal ICP waveform morphology, as well as values deemed physiologically implausible (e.g., transient extreme highs or lows indicative of noise or sensor malfunction). Following the artifact removal, the basic amplitude of the ICP pulse waveform (AMP) was calculated using discrete Fourier transform (DFT) applied to consecutive 10-s data frames with a Hanning window [29, 30]. This approach is consistent with prior work from our laboratory [31], which evaluated the effect of different windowing methods on ICP spectral estimates and demonstrated that, when analyses are performed on large aggregates of data or expressed as percentage of time above defined thresholds, the choice of windowing method has a negligible impact on the resulting calculations. ICP and ABP signals were down sampled using a 10-s non-overlapping moving average filter to highlight the slow-wave vasogenic oscillations associated with cerebral autoregulation. CPP was derived using the formula: [CPP = MAP–ICP] [1, 32, 33].

Moving Pearson correlation coefficients were calculated over 30 consecutive 10-s mean windows, updated continuously every minute, to derive CVR indices, including the pressure reactivity index (PRx: correlation between ICP and MAP), the pulse amplitude index (PAx: correlation between AMP and MAP), RAC (correlation between AMP and CPP), and the index of cerebral compensatory reserve (RAP: correlation between AMP and ICP) [1, 34, 35]. However, the overlapping nature of these windows introduced inherent autocorrelation. To address this in the ARIMA analysis, non-overlapping windows, where the data were divided into distinct, back-to-back time chunks, were used to create lower temporal resolutions, reducing autocorrelation and improving the interpretability of trend and variability measures. Finally, all data were aggregated to a minute-by-minute resolution and exported in comma-separated value (CSV) format for each patient [23].

### Data analysis

To conduct the analyses, custom Python scripts (v3.12.2, Python Software Foundation, Python Language Reference, https://www.python.org/) were developed, leveraging key libraries, such as pandas (v2.2.2), statsmodels (v0.14.2), and scikit-learn (v1.2.0). All scripts were executed on a system with an 8-core, 16-thread processor running at a base frequency of 3.8 GHz. The temporal resolution was reduced via non-overlapping averaging, starting with 1-min intervals and systematically progressing to lower resolutions, including 5-min, 10-min, 15-min, 30-min, 60-min, 2-h, 3-h, 4-h, 5-h, 6-h, 12-h, and 24-h windows. After adjusting the temporal resolution, first-order differencing was applied to address non-stationarity in the data, as identified in prior studies by our group [36]. The selection of ARIMA model parameters, autoregressive order (*p*), differencing order (*d*), and moving average order (*q*), i.e., (*p,d,q*), was guided by prior research, allowing customization for each patient and signal. Optimal values were determined by evaluating parameter ranges of 1 to 10 for *p*, 0 to 2 for *d*, and 0 to 10 for* q*, with the Akaike Information Criterion (AIC) used to identify the best fit.

To address missing data, tailored strategies were implemented to reconstruct minute-by-minute data based on the severity and extent of the data gaps. If consecutive missing values exceeded five, the corresponding data points were removed. For gaps with fewer than five missing values, interpolation was performed between the first and last available data points, with the missing values filled using the calculated interpolated values. Once preprocessed, the patient-specific signals at each temporal resolution were subjected to ARIMA analysis to generate both point and interval predictions. Because interpolation was restricted to very short gaps (< 5 consecutive timepoints) and represented a minimal portion of the total data, the impact on ARIMA model behavior is expected to be small. Short linear interpolations preserve local continuity without materially altering the broader autocorrelation patterns that drive ARIMA parameter estimation. Larger gaps were excluded to prevent distortion of temporal dynamics. Although a formal sensitivity analysis was not performed, this conservative imputation strategy minimizes the likelihood of imputation-related bias influencing model performance.

The robustness of the ARIMA models was validated by implementing multiple train/test splitting strategies, and results were assessed using standard performance metrics. In addition, the computational time required to train and test each model for every signal and temporal resolution was systematically recorded, providing insights into the efficiency of the approach.

#### ARIMA analysis

The ARIMA model is a statistical approach widely used for analyzing and forecasting time series data [21]. It is defined by three key parameters: the autoregressive (AR) order p, the differencing (integrative, I) order d, and the moving average (MA) order *q*, collectively represented as ARIMA (*p*,*d*,*q*). Each parameter captures a distinct aspect of the underlying data dynamics. p-Order reflects the number of prior observations (lags) used to predict the current value. It accounts for the relationship between the current value and its past values in the time series. Differencing (d-order) involves subtracting consecutive observations to remove trends or seasonality and achieve stationarity in the time series. The parameter *d* specifies the number of differencing steps applied to stabilize the mean and variance of the series. The parameter *q* determines the number of past error terms (residuals) used to model the current observation. It captures the influence of previous forecasting errors on the current value. A value of zero for any of these parameters implies the exclusion of the corresponding component from the model. A general form of ARIMA model is shown in Eq. 1, where Δ^*d*^*y*_*t*_ represents the differenced series, *y*_*t*_ is the observed value at time *t*, *c* denotes the constant term, *ϕ* and *θ* are the coefficients for the autoregressive and moving average components, respectively, *p* and *q* specify their respective orders, and *e* represents the lagged error terms [21, 37]:

1 $${\Delta }^{d}{y}_{t}=c+{\sum }_{i=1}^{p}{\varnothing }_{i}{y }_{t-i}+{\sum }_{j=1}^{q}{\theta }_{j}{e}_{q-j}+{e}_{t}$$

#### Data partitioning

Three distinct data partitioning methods, namely, one-step ahead cross-validation (1stepCV), blocked time-series cross validation (BlockedCV), and TimeSeriesSplit (TSS), were individually utilized for ARIMA predictions. 1stepCV approach evaluates the predictive performance of a model by iteratively forecasting one step ahead [38]. At each step, the model uses all prior observations for training and predicts the next timepoint. After each prediction, the training set is updated to include the newly observed data, allowing the model to adapt continuously. This method closely mimics the real-world forecasting scenario, where predictions are made step-by-step, making it particularly suited for time-series models that aim to provide ongoing predictions. For interval prediction, the same iterative approach is extended to forecast multiple future steps while maintaining prediction intervals. Instead of predicting only the next single value, the model forecasts multiple steps ahead in a rolling manner, generating confidence intervals for each prediction. This ensures that at each step, the model not only estimates future values but also quantifies the uncertainty associated with each forecast, providing a more comprehensive assessment of predictive reliability over a specified future horizon.

**Fig. 1:**
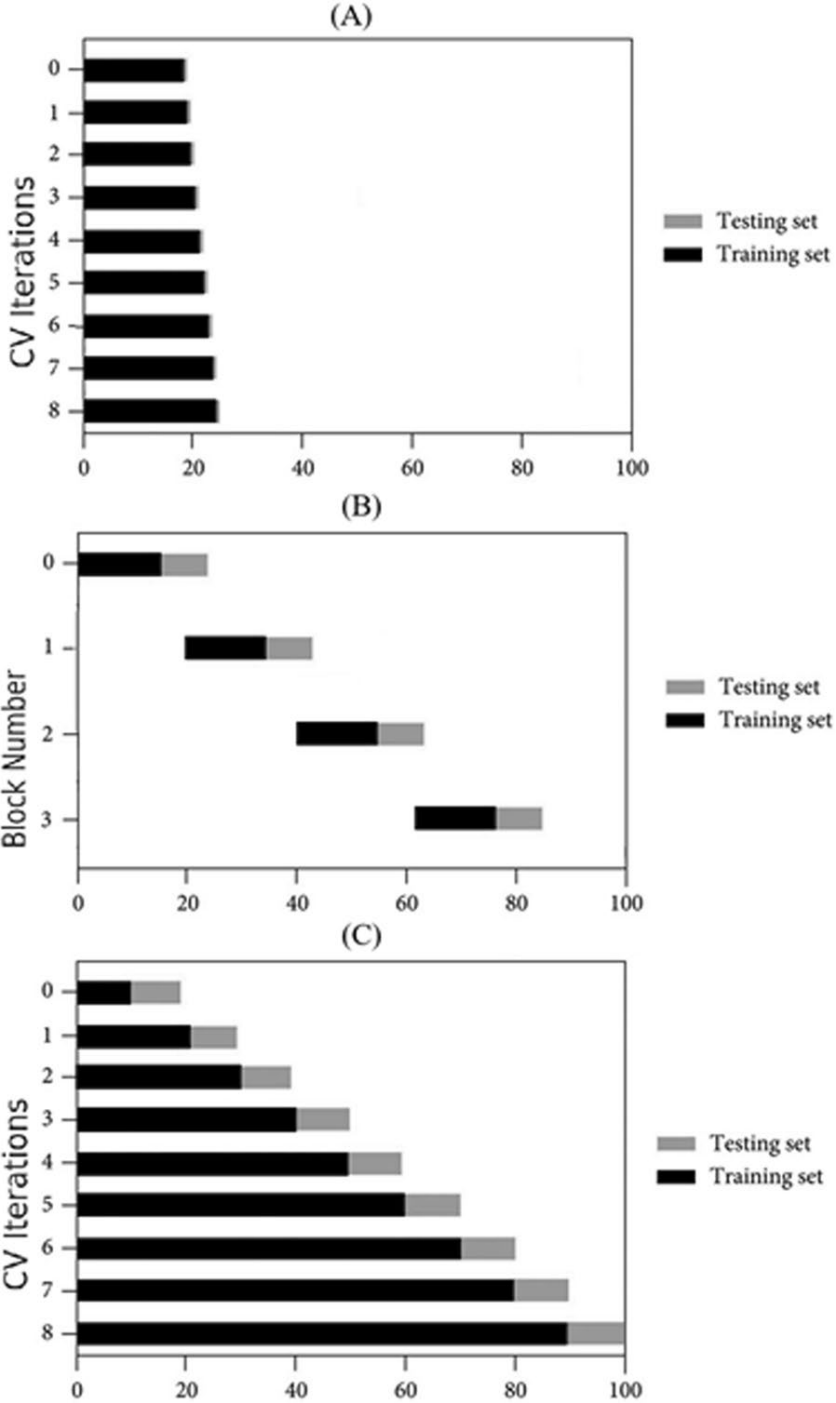
Overview of the CV approaches utilized in this study: **A** 1stepCV, sequentially training on all prior data and evaluating on the next single observation, **B** BlockedCV, featuring distinct training and testing blocks separated by gaps to avoid information leakage, and **C** TSS with progressively expanding training sets and fixed-size test sets. *1stepCV*, 1-Step Ahead Cross-Validation; *BlockedCV*, Blocked Time-Series Cross-Validation; *CV*, cross-validation; *TSS*, TimeSeriesSplit

BlockedCV is a validation method designed specifically for time-series data to address the issue of temporal dependencies. Unlike traditional CV, where data are randomly shuffled, BlockedCV divides the data into sequential, non-overlapping blocks [39]. Each fold uses one or more earlier blocks for training and a subsequent block for testing, ensuring the temporal order is maintained and future data points are not used to predict past observations. This method prevents information leakage and is particularly effective when the data exhibits strong temporal patterns or autocorrelations [40]. By preserving the natural structure of the time series, BlockedCV provides a robust approach for evaluating the performance of forecasting models while maintaining the integrity of time-dependent relationships. We created custom implementations of 1stepCV and BlockedCV. Data partitioning approaches used in this study are illustrated in Fig. 1, (A) TSS, (B) 1stepCV, and (C) BlockedCV. To balance computational efficiency with adequate training set sizes for capturing temporal patterns, the number of splits (n) was set to 5 across all methods for point prediction. This choice also aligns with standard CV practices [41]. However, BlockedCV was only employed for point-prediction and not for interval prediction due to several practical and methodological challenges. Specifically, the way BlockedCV partitions the data into fixed, non-overlapping blocks for training and testing does not align well with the requirements of interval prediction. Each fold would require careful tuning to ensure the training data adequately captures the distribution and variability needed to generate reliable prediction intervals. Even with such tuning, the resulting estimates often remained unstable or inaccurate. In addition, adapting the BlockedCV implementation for interval prediction proved to be technically problematic, frequently generating errors during model fitting and evaluation. Given the significant effort required to troubleshoot these issues with little gain in performance or reliability, we opted not to invest further time into adapting BlockedCV for interval prediction.

TSS is a cross-validation (CV) method tailored for time-series data, where the data set is split into a series of sequential train-test pairs [42]. Each split ensures that earlier timepoints are used exclusively for training and later points are reserved for testing, preserving the chronological order. Unlike standard CV, this method avoids shuffling the data, making it suitable for time-dependent models like ARIMA. With *n* splits, the training set grows incrementally with each fold, while the test set remains fixed in size, allowing the model to learn progressively more information over time without violating temporal dependencies. With *n* splits, the data set is divided into *n* + 1 sequential partitions, with each split expanding the training set by adding the next partition while preserving temporal order, allowing the model to learn progressively more information over time as the test set size remains fixed and temporal dependencies are maintained. For interval prediction, the same TSS approach is extended to forecast multiple future steps at each split. Instead of predicting a single timepoint per test set, the model generates forecasts over an interval of future points, producing corresponding confidence intervals. This method allows for both stepwise evaluation of prediction accuracy and an assessment of the uncertainty associated with forecasting over longer horizons. We employed the prebuilt implementation of TSS available in the scikit-learn library.

To prevent data leakage and overfitting, all ARIMA hyperparameters were selected independently within each cross-validation fold using only the training portion of the data, with no access to future observations. Chronological ordering was strictly preserved across 1stepCV, BlockedCV, and TSS, ensuring that test sets remained fully out-of-sample.

#### Model evaluation

Various commonly used metrics were employed to assess model accuracy, including mean absolute error (MAE), root mean square error (RMSE) and R^2^ Score (coefficient of determination), each offering distinct perspectives on the model's predictive performance [43, 44]. MAE stands out for its simplicity and interpretability, with lower values reflecting higher accuracy. MAE quantifies the average size of prediction errors, disregarding their direction. It is determined by calculating the absolute differences between predicted values and their corresponding actual values, followed by averaging these differences across all observations. Equation 2 illustrates this calculation, where *y*_*i*_ represents the actual value for the *i*th observation, *ŷ*_*i*_ is the predicted value, and *n* denotes the total number of observations:

2 $$\mathrm{MAE}=\frac{1}{n}{\sum }_{i=1}^{n}\left|{y}_{i}-{\widehat{y}}_{i}\right|$$

RMSE is another commonly used metric that calculates the square root of the average squared differences between predicted and actual values, as expressed in Eq. 3. RMSE is particularly useful when prioritizing the penalization of larger errors, as it assigns greater weight to them compared to smaller ones. In addition, RMSE is easily interpretable, since its unit matches that of the original data:

3 $$\mathrm{RMSE}=\sqrt{\frac{1}{n}{\sum }_{i=1}^{n}{\left({y}_{i}-{\widehat{y}}_{i}\right)}^{2}}$$

*R*^2^ Score is a widely used metric for evaluating the performance of regression models, measuring the proportion of variance in the dependent variable that is predictable from the independent variables. It provides an indication of how well the model captures the variability in the actual data, where an *R*^*2*^ value of 1 indicates perfect prediction, 0 indicates that the model does not do better than a simple mean-based prediction, while negative values can occur if the model performs worse than predicting the mean. On the other hand, negative values can occur when the model performs worse than predicting the mean, often due to poor fit or high noise in the data. Values exceeding 1 can arise particularly when extreme variability in the data leads to overfitting. Equation 4 illustrates the calculation of the *R*^2^ score, where ȳ is the mean of the actual values:

4 $${R}^{2}=1-\frac{{\sum }_{i=1}^{n}{\left({y}_{i}-{\widehat{y}}_{i}\right)}^{2}}{{\sum }_{i=1}^{n}{\left({y}_{i}-{\overline{y} }_{i}\right)}^{2}}$$

MAE, RMSE, and *R*^2^ were chosen as evaluation metrics as they directly assess prediction accuracy, error magnitude, and the model's ability to explain variance in the actual data, unlike metrics, such as correlation and agreement, which do not capture bias or error size as effectively in forecasting.

## Results

This study analyzed data from 376 moderate-to-severe TBI patients provided by the CAHR–TBI research collaborative. The cohort included 123 patients from the University of Calgary, 125 from the University of Manitoba, 51 from the University of Maastricht, and 77 from the University of British Columbia. Among the patients, 292 (78%) were male, with a median age of 40 years (IQR: 24–55). All patients underwent invasive monitoring of ICP and ABP, while PbtO_2_ monitoring were performed in 116 (31%) patients. Table 1 presents detailed demographic information. The study focused on individual prediction of the following physiological signals, when available, for each patient: MAP, ICP, CPP, PRx, PAx, RAC, RAP, and PbtO_2_.

**Table 1:** Demographic data

Variable	Median (Interquartile range) or Number (%)
Number of patients	376
Duration of recording (minutes)	5,904 (3,204–11,160)
Age (years)	40 (24–55)
Male sex	292 (78%)
GCS	6 (4–7)
GCS-motor	4 (1–5)
Pupils	
Bilateral reactive	246 (65%)
Bilateral unreactive	56 (16%)
Unilateral unreactive	63 (17%)
Marshall CT classification	
VI	6 (2%)
V	102 (27%)
IV	43 (11%)
III	86 (23%)
II	121 (32%)
Hypoxic episode	61 (27%)
Hypotensive episode	35 (15%)
Mean MAP (mmHg)	87 (81–93)
Mean ICP (mmHg)	12 (8–15)

*CT*, computerized tomography; *GCS*, Glasgow Coma Score; *ICP*, intracranial pressure; *MAP*, mean arterial pressure

### Signal preprocessing

Our previous study [45] evaluated the stationarity of the original physiological signals using the Augmented Dickey–Fuller (ADF) and Kwiatkowski–Phillips–Schmidt–Shin (KPSS) tests. The KPSS test consistently indicated non-stationarity, while the ADF test showed that non-stationarity increased as the temporal resolution decreased, highlighting the need for first-order differencing. This trend underscores the challenges of applying ARIMA models at lower temporal resolutions, where the reduction in data points and the loss of finer temporal details due to point-sampling can hinder the determination of optimal model parameters. To mitigate these issues, first-order differencing was applied to all signals following temporal resolution reduction, ensuring stationarity and improving the data's suitability for ARIMA modeling.

The stationarity analysis revealed inconsistencies between the ADF and KPSS tests, with KPSS consistently indicating non-stationarity, while ADF classified most signals as stationary. However, as temporal resolution decreased, ADF also detected non-stationarity, reflecting challenges in applying ARIMA models due to fewer data points and trends introduced by larger averages. To assess and address autocorrelation in the ICP signal, autocorrelation function (ACF) and partial autocorrelation function (PACF) analyses were performed on a 5-min average-sampled signal from a representative patient. Prior to ARIMA modeling, the ACF and PACF plots showed substantial autocorrelation, with multiple lags exceeding the confidence intervals. After fitting the ARIMA model with the optimized parameters, the residual autocorrelation was notably reduced, indicating improved model performance. The corresponding ACF and PACF plots, both before and after ARIMA application for an example case, are presented in Fig. A. 1 in the supplementary file. The median ARIMA parameters (optimized via AIC) for each physiological signal and temporal resolution employed in this study are also listed in the supplementary file in Table A. 1. In general, the p-order ranged from 2 to 5, while lower temporal resolutions often required second-order differencing (*d*-order = 2).

### ARIMA model performance

The performance of ARIMA models was systematically evaluated for each physiological signal across various temporal resolutions. This evaluation included generating both point predictions and prediction intervals to assess the models’ accuracy and reliability. The results of the point predictions were evaluated using metrics, such as MAE, RMSE, and R^2^ score, providing quantitative measures of prediction accuracy and model performance. For interval predictions, the CI results were also presented, highlighting the range within which the true values are likely to fall with a specified level of certainty.

#### Predictive performance across temporal resolutions with point prediction

In point prediction, the predictive performance across temporal resolutions, evaluated through MAE, RMSE, and R^2^ metrics, showed consistent trends across signals, with similar outcomes regardless of the choice of data partitioning method. To ensure sufficient training data for capturing temporal patterns while maintaining computational efficiency, the number of splits (*n*) was fixed at 5 for all methods.

Overall, the results show a clear trend in predictive accuracy of ARIMA across temporal resolutions. At high resolutions (e.g., 1 min and 5 min), ARIMA performs well, particularly for CVR metrics (PRx, PAx, RAC and RAP), with MAE values below 0.2 across all data partitioning methods, i.e., 1stepCV, BlockedCV and TSS. However, as the resolution decreases, MAE values increase, particularly for raw signals such as MAP, CPP, ICP, and PbtO_2_ with MAP and CPP peaking above 7 at 720 min and 1440 min. While 1stepCV shows a steep rise in MAE at lower resolutions, BlockedCV exhibits a slightly less pronounced increase, especially for CPP and MAP, indicating potential robustness for long-term forecasting. TSS results closely align with the other two methods but display smoother MAE transitions, likely due to its progressive training approach.

RMSE results follow similar trends to MAE, with raw signals such as CPP, ICP, and MAP showing notably higher errors at lower resolutions (e.g., 720 min and 1440 min), often exceeding 10, indicating substantial forecasting challenges. In contrast, CVR metrics (PAx, PRx, and RAC) maintain lower RMSE values, suggesting more stable patterns. R^2^ scores further highlight these trends, with near-zero values at high resolutions (1 min to 10 min), slight improvements at intermediate resolutions (30 min to 120 min), and sharp declines at lower resolutions, particularly for CPP, and MAP. At 1440 min, ICP, MAP, and PbtO_2_ show poor fitting due to fewer data points and increased noise sensitivity, as seen in their *R*^*2*^ values dropping well below 0. Meanwhile, CVR-derived metrics maintain more stable *R*^*2*^ and MAE values, likely due to their derivation from correlations rather than direct physiologic measurements, making them less susceptible to increased variability at lower resolutions.

**Fig. 2:**
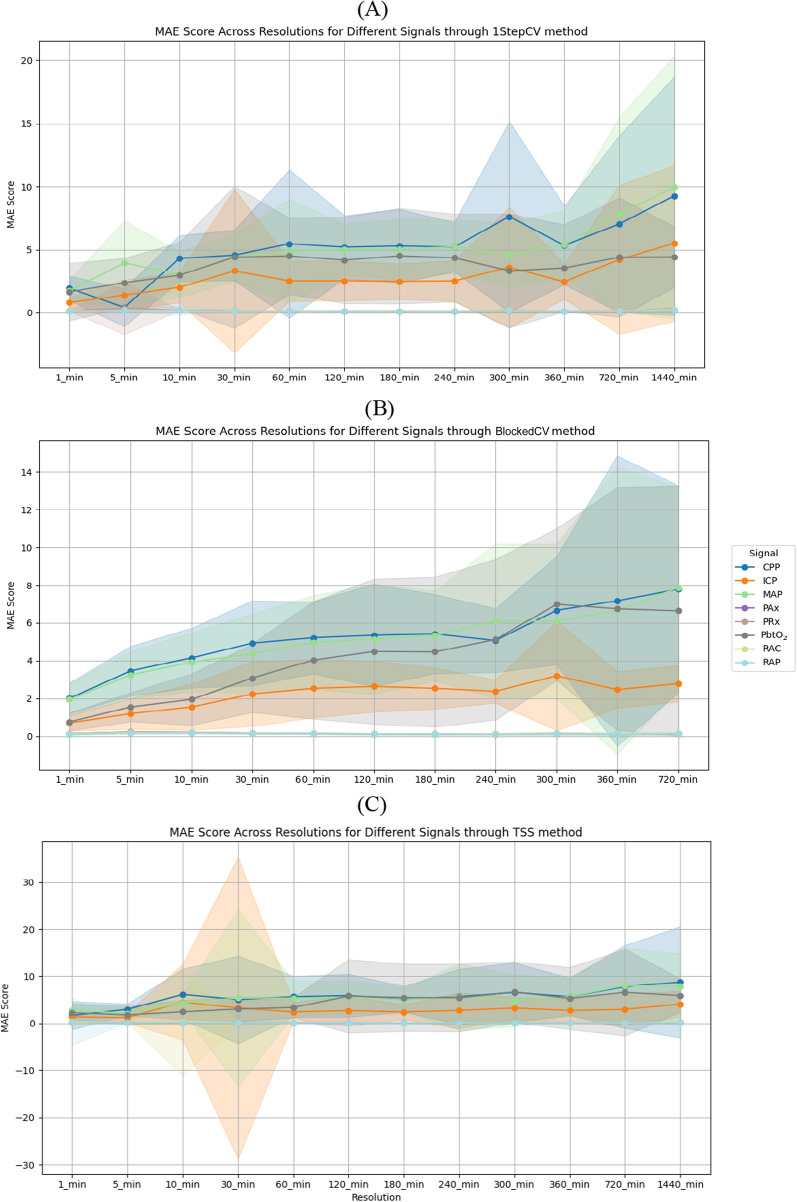
Line graphs of the MAE across different cerebral physiological signals and temporal resolutions using **A** 1stepCV, **B** BlockedCV, and **C** TSS for point prediction. Solid lines show mean MAE, and shaded regions indicate the standard deviation across cross-validation folds. Note that while the shaded areas may extend visually below zero due to standard deviation plotting, MAE values themselves are strictly non-negative. *1stepCV*, 1-step cross validation; *BlockedCV*, Blocked Time-Series Cross-Validation; *CPP*, cerebral perfusion pressure; *ICP*, intracranial pressure; *MAE*, mean absolute error; *MAP*, mean arterial pressure; *PAx*, pulse amplitude index; *PbtO*_*2*_, cerebral oxygen saturation; *PRx*, pressure reactivity index; *RAP*, index of cerebral compensatory reserve; *TSS*, TimeSeriesSplit

The MAE trends across CV methods (1stepCV, BlockedCV, and TSS) show a general increase in error as temporal resolution decreases, highlighting the challenges of long-term forecasting. In 1stepCV (Fig. 2A), MAE remains low for short intervals (1 min to 10 min) but rises significantly at lower resolutions, especially for CPP, MAP, ICP, and PbtO_2_, i.e., raw physiological signals. BlockedCV (Fig. 2B) follows a similar trend but with greater variability across signals, suggesting raw physiological signals are more affected by this method than derived metrics, such as PAx and PRx. TSS (Fig. 2C) shows an inconsistent MAE pattern with large fluctuations at certain resolutions, indicating ARIMA struggles with non-overlapping time segments. Overall, all methods show increasing MAE at lower resolutions, with BlockedCV introducing more variability and TSS exhibiting greater fluctuations.

**Fig. 3:**
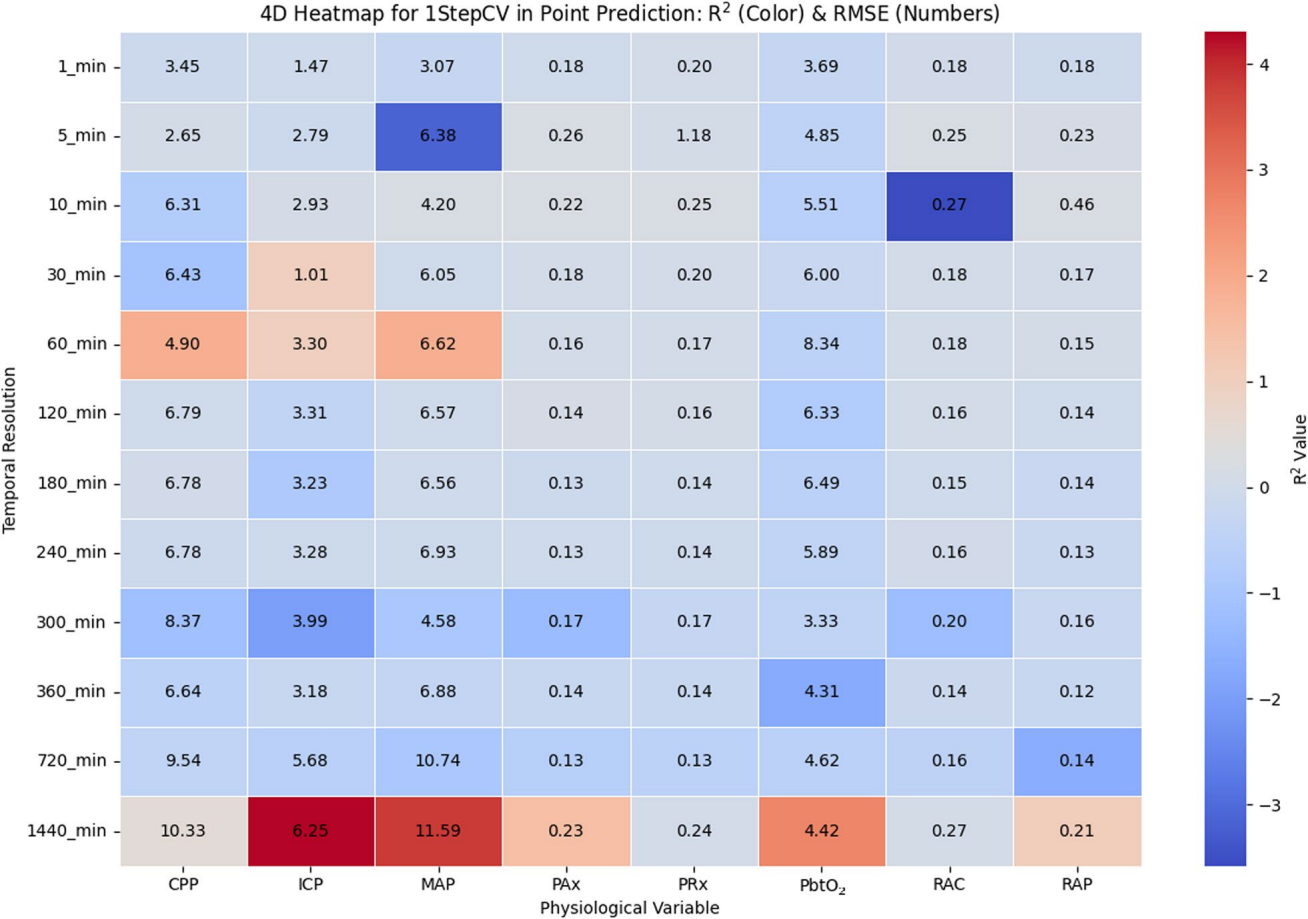
Heatmap illustrating the relationship between temporal resolution and physiologic variables in ARIMA point prediction for 1stepCV. The color gradient represents the *R*^*2*^ values, indicating model fit quality, while the numeric values in each cell denote the RMSE, reflecting prediction error. R^2^ and RMSE are calculated separately for each patient at each temporal resolution, and the values displayed represent the mean across all patients. R^2^ and RMSE are computed separately for each patient at each temporal resolution, and the values shown represent the mean across all patients. *1stepCV*, 1-step cross validation; *CPP*, cerebral perfusion pressure; *ICP*, intracranial pressure; *MAP*, mean arterial pressure; *PAx*, pulse amplitude index; *PbtO*_*2*_, cerebral oxygen saturation; *PRx*, pressure reactivity index; *R*^*2*^, R^2^ score; *RAP*, index of cerebral compensatory reserve; *RMSE*, root mean square error

The four-dimentional (4D) heatmap given in Fig. 3 illustrates the relationship between temporal resolution, physiological variables, *R*^*2*^ values shown as the colors, and RMSE values shown as the numbers, providing insight into the performance of ARIMA-based point predictions with 1stepCV. Overall, *R*^*2*^ values tend to decrease as temporal resolution increases, indicating that ARIMA models become less effective in prediction at lower resolutions. However, certain physiological variables exhibit relatively stable *R*^*2*^ values across resolutions, suggesting they may have more predictable trends. The RMSE values further support this trend, with lower errors observed at higher temporal resolutions (1 min to 10 min), where short-term autoregressive dependencies are more effectively captured. On the other hand, RMSE increases significantly at lower resolutions (≥ 60 min), highlighting the difficulties of prediction at lower resolutions. In addition, certain cerebral physiological variables, such as ICP and MAP, demonstrate strong degradation in *R*^*2*^ values with decreasing temporal resolution, suggesting that their fluctuations are less predictable at lower resolutions. In contrast, some indices, such as PRx and PAx, show moderate predictability even at these lower resolutions.

**Fig. 4:**
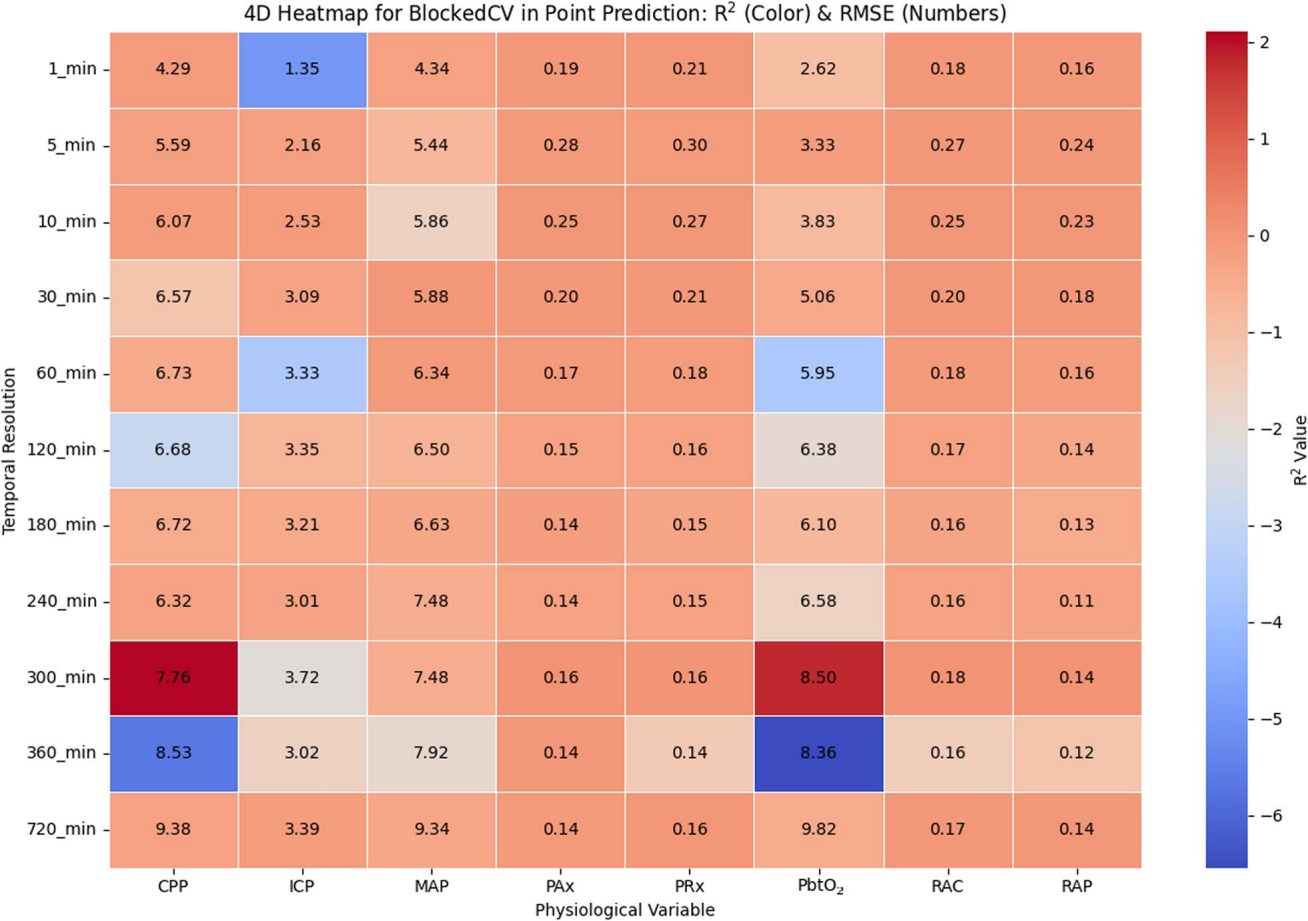
Heatmap illustrating the relationship between temporal resolution and physiologic variables in ARIMA point prediction for BlockedCV. The color gradient represents the *R*^*2*^ values, indicating model fit quality, while the numeric values in each cell denote the RMSE, reflecting prediction error. R^2^ and RMSE are computed separately for each patient at each temporal resolution, and the values shown represent the mean across all patients. *BlockedCV*, Blocked Time-Series Cross-Validation; *CPP*, cerebral perfusion pressure; *ICP*, intracranial pressure; *MAP*, mean arterial pressure; *PAx*, pulse amplitude index; *PbtO*_*2*_, cerebral oxygen saturation; *PRx*, pressure reactivity index; *R*^*2*^, R^2^ score; *RAP*, index of cerebral compensatory reserve; *RMSE*, root mean square error

Compared to the 1-stepCV, the BlockedCV method exhibits a generally similar pattern in *R*^*2*^ values, as illustrated in Fig. 4, with better predictability at higher temporal resolutions and a decline as the resolution decreases. However, the BlockedCV method appears to yield slightly higher *R*^*2*^ values at higher temporal resolutions (e.g., 5 min to 30 min), suggesting that blocking the training and test sets reduces data leakage and improves model generalizability at higher resolutions. This is particularly evident in variables, such as MAP and PRx, where BlockedCV results in more stable *R*^*2*^ values across different resolutions. An important difference is that the RMSE values in BlockedCV tend to be slightly higher for several cerebral physiological variables, particularly at lower resolutions (60 min and lower). Notably, the BlockedCV method shows clearer fluctuations in *R*^*2*^ values for certain variables such as PAx and PRx at lower resolutions (above 300 min). This could indicate that blocking introduces additional variance in the model’s performance due to temporal dependencies being broken across blocks. In addition, ICP and MAP exhibit higher RMSE values in BlockedCV than in 1stepCV.

**Fig. 5:**
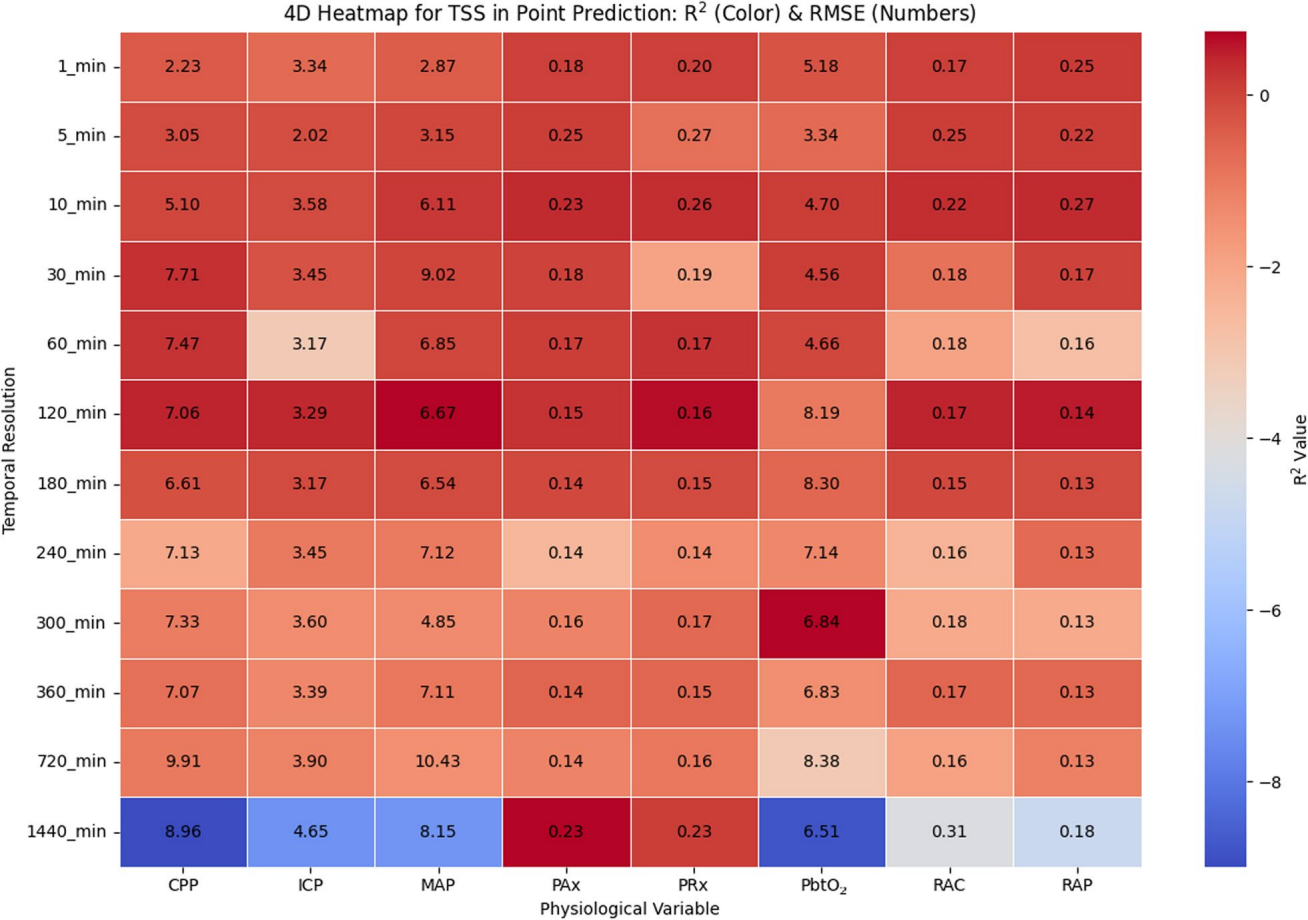
Heatmap illustrating the relationship between temporal resolution and physiologic variables in ARIMA point prediction for TSS. The color gradient represents the *R*^*2*^ values, indicating model fit quality, while the numeric values in each cell denote the RMSE, reflecting prediction error. R^2^ and RMSE are computed separately for each patient at each temporal resolution, and the values shown represent the mean across all patients. *CPP*, cerebral perfusion pressure; *ICP*, intracranial pressure; *MAP*, mean arterial pressure; *PAx*, pulse amplitude index; *PbtO*_*2*_, cerebral oxygen saturation; *PRx*, pressure reactivity index;* R*^*2*^, R^2^ score; *RAP*, index of cerebral compensatory reserve; *TSS*, TimeSeriesSplit

The TSS method produced results that share similarities with both 1stepCV and BlockedCV but also show distinct differences, as shown in 4D heatmap in Fig. 5. Similar to the other methods, TSS demonstrates relatively higher *R*^*2*^ values at higher temporal resolutions (below 10 min), with a progressive decline as the resolution decreases. This suggests that short-term predictions remain feasible, but ARIMA struggles with long-term forecasting regardless of the cross-validation method. Compared to BlockedCV, TSS tends to yield slightly higher *R*^*2*^ values across most temporal resolutions, particularly in signals, such as CPP and MAP. This could be attributed to the overlapping nature of training and test sets in TSS allowing the model to better capture autoregressive patterns and maintain continuity in trends. However, this also introduces a risk of data leakage, which may artificially inflate performance estimates compared to the more conservative BlockedCV approach. A notable difference from both previous methods is observed at the lowest resolutions (720 min and 1440 min), where TSS results in more extreme variations in *R*^*2*^ values, including highly negative values specifically for raw cerebral physiological signals, such as ICP, MAP, and PbtO_2_.

To assess whether ARIMA forecasting performance varied across clinical subgroups, we performed statistical comparisons of MAE using two-sample *t* tests for binary variables (age group (> = 40 vs. < 40), Marshall CT score (> = 5 vs. < 5), and sex (male vs. female) and a one-way ANOVA for the three-level pupillary reactivity classification (bilaterally reactive, bilaterally unreactive vs unilateral unreactive). These analyses were conducted separately for each temporal resolution and for each data splitting method. Across all tested subgroups and resolutions, no statistically significant differences (p > 0.05) were found, suggesting that subgroup characteristics did not meaningfully influence ARIMA prediction accuracy under the examined conditions. The extended results are presented in Supplementary Table A. 2.

#### Predictive performance across temporal resolutions with interval prediction

The predictive performance of ARIMA models for interval prediction, evaluated through MAE, RMSE, and R^2^ score across temporal resolutions, exhibited distinct patterns across signals, with variations depending on the choice of CV strategy. The interval was set as 5 for both 1stepCV and TSS to balance accuracy and computational cost, ensuring a sufficient number of future points were predicted while maintaining feasible model training times.

MAE variations for different physiological signals across temporal resolutions using the 1stepCV method show that errors remain low at higher resolutions but increase for signals such as CPP, MAP, and PbtO_2_ at lower resolutions. PbtO_2_, in particular, exhibits the sharpest rise at 720 min and 1440 min, indicating challenges in long-term forecasting. Similarly, the TSS method follows this trend but with a more gradual increase in MAE, suggesting greater stability due to its multiple training and testing windows. Notably, signals such as PbtO_2_ and MAP still exhibit the highest errors at lower resolutions. Compared to 1stepCV, TSS yields slightly lower MAE, especially at mid-range resolutions (120 min to 360 min), highlighting its ability to capture longer term trends while minimizing data leakage. Overall, while short-term forecasting is more accurate, long-term predictions introduce greater variability, particularly for MAP and PbtO_2_. For more details, MAE, RMSE and R^2^ score heatmaps of both 1stepCV and TSS are provided in the supplementary file from Figure A. 11 to Figure A. 16.

The R^2^ heatmaps for 1stepCV and TSS reveal declining predictive performance of ARIMA model at lower temporal resolutions, consistent with MAE and RMSE trends. Raw signals such as CPP, ICP, MAP, and PbtO_2_ show the lowest *R*^*2*^ values at 720 min and 1440 min, highlighting long-term forecasting challenges. TSS maintains higher R^2^ scores at mid-range resolutions (120 min to 360 min) compared to 1stepCV, suggesting better handling of long-term dependencies. However, at the lowest resolutions, both methods see a decline, particularly for PbtO_2_ and MAP, with 1stepCV showing a sharper drop, while TSS declines more gradually.

These findings indicate that raw cerebral physiologic signals exhibit greater long-term variability, that ARIMA struggles to model effectively, compared to derived signals, such as PRx, PAx, etc. In short, TSS appears to provide a more robust validation approach for mid-range interval forecasting, as it preserves temporal dependencies and mitigates the sharp performance degradation seen in 1stepCV. However, at the highest temporal resolutions, both methods struggle to produce meaningful predictions, particularly for raw signals, suggesting that alternative modeling approaches may be necessary for long-term forecasting.

In addition, the analysis of CI spread across different temporal resolutions using ARIMA also reveals a clear trend of increasing uncertainty with longer forecast horizons. As the temporal resolution decreases, the CI spread generally widens, indicating higher forecast uncertainty. A comparison between 1stepCV and TSS shows similar trends in CI spread across resolutions, presented in the supplementary file from Figure A. 17 to Figure A. 20. However, the TSS method tends to exhibit slightly larger variance at lower resolutions, suggesting increased prediction uncertainty when using this validation approach. The CI spread is relatively low at the highest temporal resolution (1 min), indicating more reliable short-term predictions. However, at the lowest resolution (1440 min), while the CI spread appears smaller compared to mid-range resolutions, this result is likely biased due to the significant decrease in available data points at this resolution. The reduced data may lead to an artificial narrowing of the confidence intervals rather than an actual improvement in forecast reliability. Therefore, the results at 1440-min resolution should not be heavily considered when interpreting overall trends.

In addition, the presence of outliers is observed in both validation approaches, indicating occasional high-uncertainty predictions. However, these findings highlight that short-term predictions using ARIMA are generally more reliable, and that the choice of CV strategy also influences the degree of forecast uncertainty, with TSS leading to slightly wider intervals at lower resolutions.

**Fig. 6:**
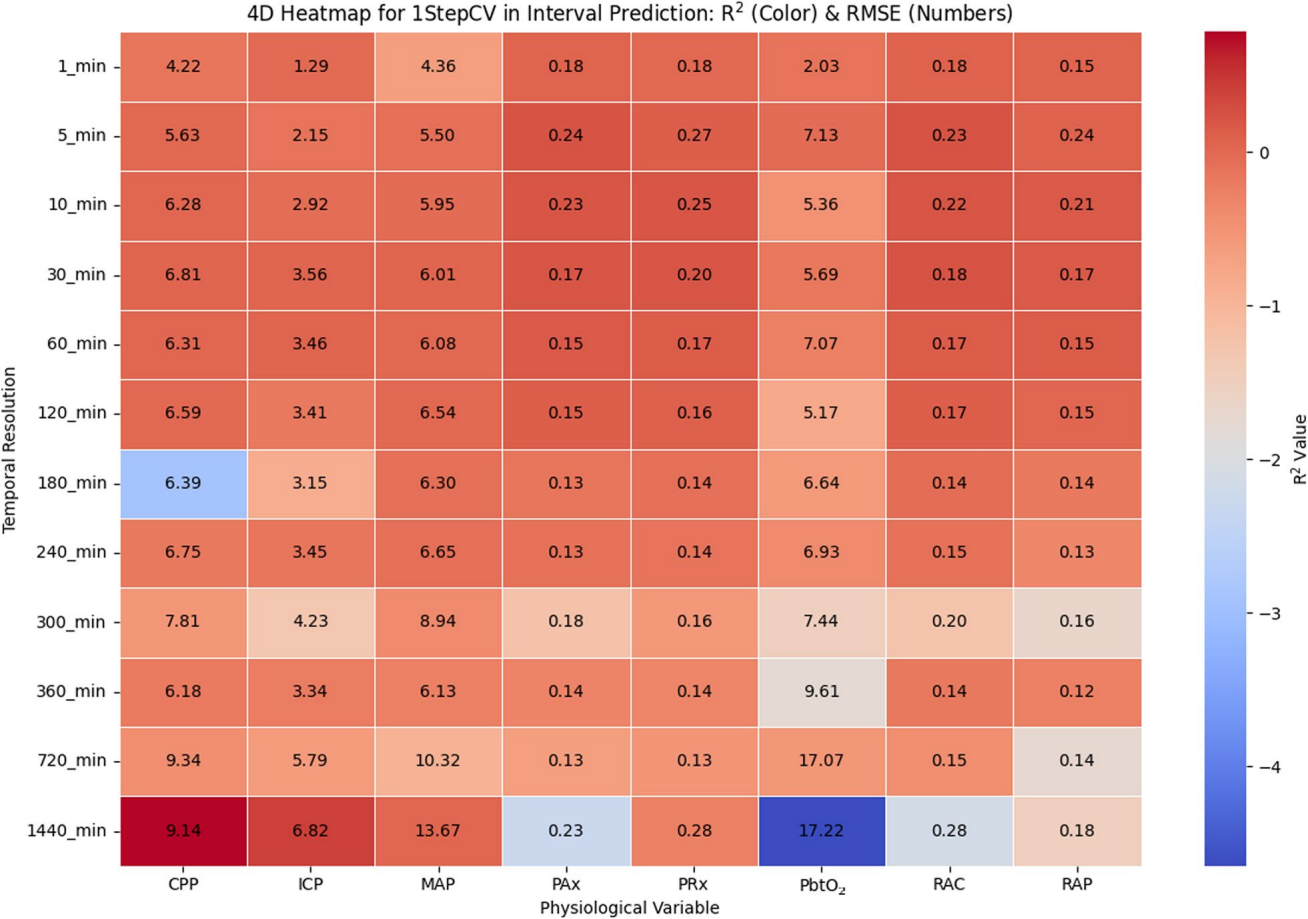
Heatmap illustrating the relationship between temporal resolution and physiologic variables in ARIMA interval prediction for 1stepCV. The color gradient represents the *R*^*2*^ values, indicating model fit quality, while the numeric values in each cell denote the RMSE, reflecting prediction error. R^2^ and RMSE are computed separately for each patient at each temporal resolution, and the values shown represent the mean across all patients. *1stepCV*, 1-step cross validation; *CPP*, cerebral perfusion pressure; *ICP*, intracranial pressure; *MAP*, mean arterial pressure; *PAx*, pulse amplitude index; *PbtO*_*2*_, cerebral oxygen saturation; *PRx*, pressure reactivity index;* R*^*2*^, R^2^ score; *RAP*, index of cerebral compensatory reserve; *RMSE*, root mean square error

The 4D heatmap for the 1stepCV interval prediction presented in Fig. 6 highlights key trends in model performance across different temporal resolutions and physiological variables. Overall, the *R*^*2*^ values remain relatively low throughout, with most staying near or below 0.2. This suggests that the interval prediction method struggles to establish strong predictive relationships within the data. Certain physiological variables, such as PbtO_2_, display extreme variations at lower temporal resolutions, with some values turning significantly negative. These negative *R*^*2*^ values indicate that the model performs worse than a simple predictor, suggesting poor fit in these cases. When analyzing performance across different temporal resolutions, highest resolutions (below 30 min) show consistently low *R*^*2*^ values, though RMSE remains relatively moderate. As resolution decreases (from 60 to 360 min), RMSE values gradually rise, reflecting a steady increase in prediction error. At the lowest resolutions (720 min and 1440 min), *R*^*2*^ values become more unstable, particularly for PbtO_2_, where extreme negative values indicate that the model fails to generalize well over longer forecasting horizons.

The RMSE values reveal further insights into prediction performance. As the temporal resolution decreases, RMSE values show a noticeable upward trend, particularly for variables, such as CPP, ICP, and MAP, which suggests growing absolute errors in interval predictions over longer time intervals. In contrast, some physiological variables, such as PAx and PRx, maintain relatively stable RMSE values across different resolutions, indicating less variability in prediction error for these signals. Comparing performance across physiological variables, some signals, such as PRx and RAP, demonstrate relatively stable *R*^*2*^ values across temporal scales, whereas others, particularly PbtO_2_, exhibit drastic performance drops at lower resolutions. PRx and RAP maintain relatively stable *R*^*2*^ values but still experience increasing RMSE as the temporal resolution decreases, reflecting a growing deviation from observed values.

**Fig. 7:**
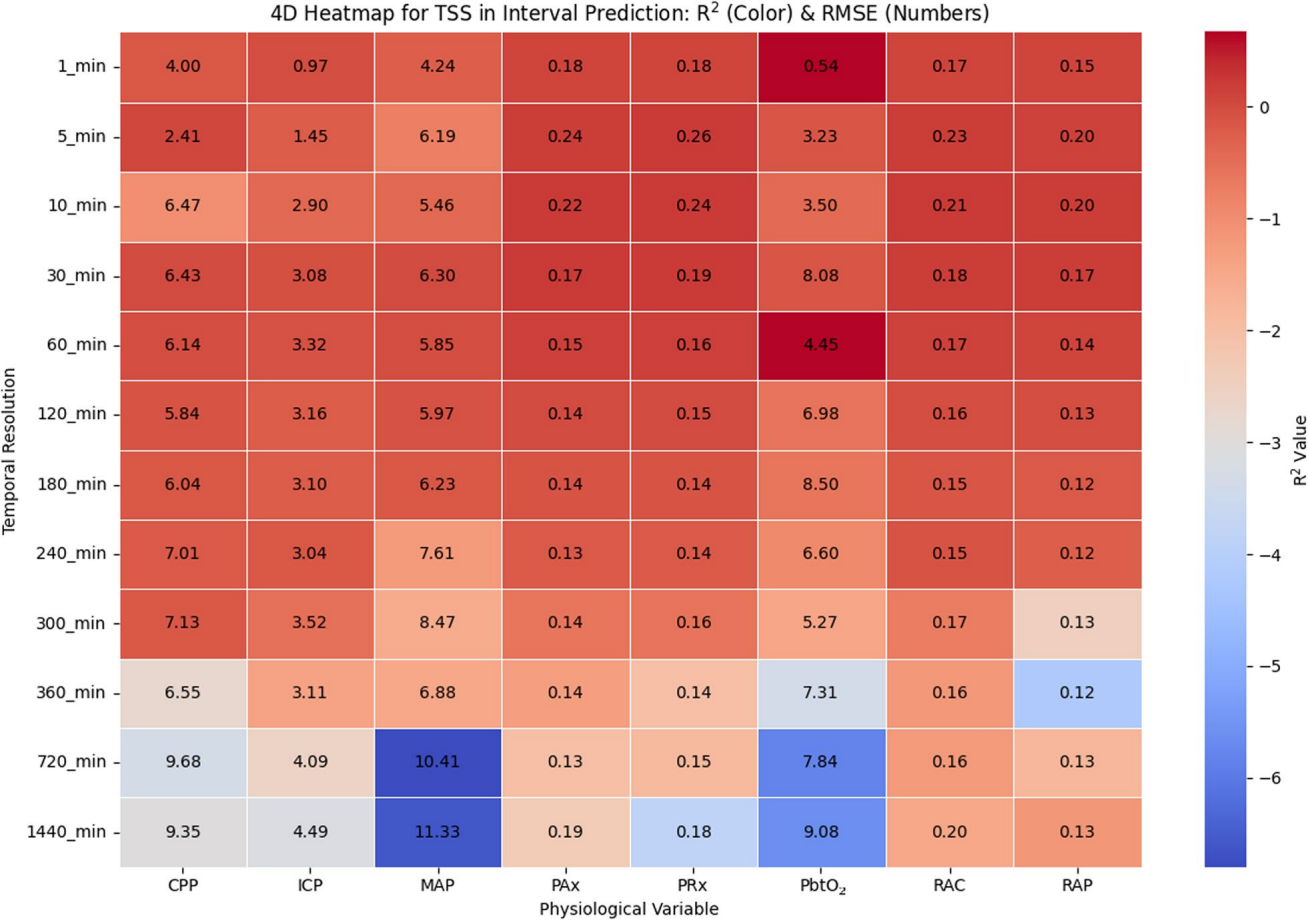
Heatmap illustrating the relationship between temporal resolution and physiologic variables in ARIMA interval prediction for TSS. The color gradient represents the *R*^*2*^ values, indicating model fit quality, while the numeric values in each cell denote the RMSE, reflecting prediction error. R^2^ and RMSE are computed separately for each patient at each temporal resolution, and the values shown represent the mean across all patients. *CPP*, cerebral perfusion pressure; *ICP*, intracranial pressure; *MAP*, mean arterial pressure; *PAx*, pulse amplitude index; *PbtO*_*2*_, cerebral oxygen saturation; *PRx*, pressure reactivity index;* R*^*2*^, R^2^ score; *RAP*, index of cerebral compensatory reserve; *RMSE*, root mean square error; *TSS*, TimeSeriesSplit

When comparing the TSS interval prediction results, as shown in Fig. 7, to the 1stepCV approach, several key differences emerge in terms of model performance across temporal resolutions and cerebral physiological variables. Overall, the TSS method exhibits slightly more stable *R*^*2*^ values than the 1stepCV approach, although both models struggle with predictive accuracy, as indicated by consistently low *R*^*2*^ values. Similar to 1stepCV, TSS fails to capture strong predictive relationships across most signals, but extreme negative *R*^*2*^ values are less pronounced, particularly at lower temporal resolutions. In terms of RMSE trends, both methods show increasing error as temporal resolution decreases. However, in TSS, RMSE values for variables such as MAP, ICP, and CPP rise even more dramatically at lower resolutions (720 min and 1440 min), with MAP showing the highest RMSE among all signals. In contrast, 1stepCV displayed more variable R^2^ behavior at lower resolutions, particularly with PbtO_2_.

Overall, neither method achieves strong predictive performance, but TSS appears to provide slightly better stability in terms of *R*^*2*^ values, whereas 1stepCV struggles more with extreme negative values at lower resolutions. However, TSS exhibits higher RMSE values for certain signals like MAP, indicating that while it shows predictive relationships better, its absolute prediction errors are substantial. These findings suggest that both methods may require further refinement to improve interval prediction accuracy across different physiological signals and temporal scales.

#### Computational times

The computational times for training and testing the ARIMA model varied significantly based on the CV method, prediction type, i.e., point or interval, and signal analyzed. Training times ranged from 30 s to a-minute with 1stepCV method on point prediction, while testing times stayed around 0.15 s. For the TSS method applied to point predictions, training times ranged widely, from minute to an hour in average. Testing times, however, were consistently faster, generally staying below 1 s for most signals. In BlockedCV for point prediction, the training times for most signals hovered around 0.5–2 s per block, with testing times consistently remained under 0.01 s.

For interval predictions using the 1stepCV method, training times ranged from 37 to 1400 s. The TSS method exhibited training times as high as 565 s. Testing times for both methods were typically below 1 s on average. For all methods employed, regardless of prediction type, training times dropped substantially, reaching durations in the range of tens of seconds or less, as the temporal resolution decreased. The median computational times for training and testing under various configurations are tabulated in Table A. 3 in the supplementary file, highlighting the differences between point and interval predictions and CV strategies. Importantly, variations in computational times between signals primarily reflect differences in data volume and signal-specific characteristics.

## Discussion

In this study, we evaluated the predictive performance of ARIMA models on raw and derived cerebral physiologic signals under mean-averaged temporal resolution reduction, considering both point and interval predictions. We employed three CV methods suitable for time-series data to assess variations in results based on the chosen method. The performances were evaluated using MAE, RMSE, and *R*^2^ scores, along with CI for interval predictions.

The findings of point prediction highlight the impact of temporal resolution and data partitioning methods on ARIMA-based forecasting of cerebral physiological signals. The findings reveal that ARIMA models perform optimally at higher temporal resolutions (e.g., 1-min and 5-min intervals), particularly for CVR indices, such as PAx, PRx, and RAC. The relatively low MAE and RMSE values for these metrics suggest that their structured and stable nature allows for more accurate forecasting. On the other hand, raw physiological signals, including ICP, CPP, and MAP, exhibit significant increases in MAE and RMSE at lower temporal resolutions (e.g., 720-min and 1440-min intervals). This trend suggests that these signals are more susceptible to data loss and increased complexity when down-sampled, leading to reduced predictive accuracy. The decline in *R*^2^ scores at lower resolutions further reinforces ARIMA models’ struggle to capture long-term trends effectively. The sharp decrease in *R*^*2*^ values for signals such as MAP and CPP at lower temporal resolutions highlights the increasing difficulty in modeling these signals using standard time-series approaches. This is likely due to the increased variability and reduced predictive structure introduced at lower temporal resolutions, despite differencing being applied to address non-stationarity.

Comparing the three CV methods (1stepCV, BlockedCV, and TSS), the results demonstrate that each approach influences predictive performance differently. The 1stepCV method generally produces lower MAE values at higher temporal resolutions, making it well-suited for short-term forecasting tasks. However, as the resolution decreases, MAE values increase, particularly for raw physiological signals, indicating diminishing predictive accuracy. While BlockedCV, which enforces strict temporal ordering in training and testing, introduces slightly higher MAE values at high resolutions compared to 1stepCV but demonstrates more robustness at lower resolutions. This suggests that while BlockedCV captures temporal dependencies more realistically, it also introduces greater variability in error estimates, particularly for raw signals, such as CPP, ICP, and MAP. TSS, on the other hand, exhibits a more inconsistent pattern in MAE trends, with larger fluctuations observed across temporal resolutions. This variability indicates that ARIMA models may struggle to maintain stable predictive performance when trained on progressively expanding subsets of the data. Despite that, TSS appears to be more forgiving than BlockedCV in terms of generalization across different temporal resolutions, as indicated by slightly lower MAE values at lower resolutions.

The observed differences in predictive performance across signals and CV methods have important implications for time-series modeling in cerebral physiology. The consistently lower prediction errors for CVR indices across resolutions suggest that these metrics may be more suitable for ARIMA-based forecasting, particularly in applications requiring real-time or short-term predictions. In contrast, the higher MAE and RMSE values observed for raw signals at lower resolutions highlight the need for alternative modeling strategies, such as incorporating external covariates, using machine learning-based forecasting models, or leveraging hybrid approaches that integrate ARIMA with deep learning techniques. Furthermore, the variability introduced by different CV methods suggests that model evaluation strategies should be carefully selected based on the forecasting objective. While 1stepCV provides a more optimistic estimate of model performance, BlockedCV offers a more realistic assessment of long-term forecasting ability. TSS, despite its fluctuations, presents a feasible middle ground, balancing short-term and long-term prediction performance.

Similarly, the results for internal prediction demonstrated that ARIMA models show variable predictive performance across different physiological signals, temporal resolutions, and CV strategies. Predictions at higher temporal resolutions performed well with relatively low error rates, while longer forecasting horizons introduced higher uncertainty, especially for raw signals, such as MAP, PbtO_2_, and CPP. Similar trend to point prediction is observed, where MAE increases notably at lower resolutions, particularly for raw cerebral physiologic signals, specifically PbtO_2_ which experiences a sharp rise in MAE at the lowest resolutions, indicating poor long-term predictability. Derived signals, such as PRx and PAx, exhibit more stable predictions over time, suggesting they are easier to forecast than raw signals.

Comparing the two data partitioning methods show that TSS outperforms 1stepCV, offering lower MAE and higher *R*^*2*^ values at mid-range resolutions (120–360 min), likely due to its ability to capture longer term trends. However, both strategies struggle at the lowest resolutions (720 min and 1440 min), where predictive accuracy decreases sharply. In addition, CI show wider spreads at lower resolutions, reflecting higher uncertainty in long-term predictions. The CI is narrower at the highest resolution (1 min), indicating more reliable short-term forecasts. However, it is important to note that at 1440-min resolution, the reduced number of data points may artificially narrow the CI, leading to potentially misleading results.

In addition, computational times for ARIMA model training and testing varied depending on the data partitioning method, prediction type, and signal. Point prediction with 1stepCV had short training times (30 s to a minute per fold) and fast testing (< 0.15 s), while TSS required longer training (1–60 min per fold) with quick testing time (< 1 s). However, BlockedCV was the most efficient, with short training (0.5–2 s per block) and very fast testing (< 0.01 s). For interval predictions, training times were much longer, with 1stepCV ranging from 37 to 1400 s and TSS up to 565 s per fold. Testing times were consistently below 1 s for both methods. Regardless of prediction type, training times decreased as temporal resolution lowered, due to shrinking data set sizes.

The differences in model performance across temporal resolutions carry important clinical implications. Short-term forecasts (e.g., 1–15 min) may support real-time bedside decisions, such as anticipating imminent ICP elevations before they occur, prompting earlier administration or adjustment of hyperosmolar therapy, or guiding rapid titration of vasopressor support to maintain optimal CPP [46, 47]. Short-term predictions of PbtO_2_ or compliance could similarly help clinicians identify evolving hypoxic or low-compliance states and intervene before deterioration becomes clinically apparent [48, 49]. In contrast, longer term predictions (e.g., 6 h and longer) may inform broader management strategies, including planning the timing of follow-up CT imaging, anticipating the trajectory of intracranial hypertension or autoregulatory status over the next shift, guiding sedation tapering plans, or identifying when patients may be transitioning from acute instability toward a more stable phase of care [50, 51]. Hence, forecasting of cerebral physiology at different temporal scales could complement existing neuromonitoring tools and support both immediate and anticipatory clinical decision-making.

In short, this study underscores the importance of temporal resolution and data partitioning methods in ARIMA-based forecasting of cerebral physiological signals. While ARIMA models perform well at high temporal resolutions, their effectiveness declines as resolution decreases, especially for raw physiological signals. The choice of CV method also affects performance, with BlockedCV showing more variability and TSS providing more stable predictions at mid-range resolutions. However, both methods face challenges at low resolutions, suggesting the need for alternative modeling approaches for long-term forecasting. In addition, while short-term ARIMA predictions are generally reliable, longer term forecasts are more computationally demanding, particularly with more complex methods like TSS. The overall results highlight the need for further exploration of hybrid approaches to improve long-term prediction reliability in cerebral physiology signals, specifically raw signals, such as ICP, CPP, MAP, and PbtO_2_.

## Limitations

A key limitation of this study stems from the fundamental assumptions of the ARIMA model. As a linear model, ARIMA may struggle to fully capture the complexity and non-linearity present in certain physiological signals, particularly those with high variability, such as MAP and CPP. These signals, which can exhibit sudden fluctuations due to physiological changes, may be challenging for ARIMA to model effectively, especially at lower temporal resolutions. Alternative approaches, such as non-linear models or hybrid techniques, could provide better predictive performance by accounting for more intricate patterns and dependencies in the data.

Another limitation lies in the assumptions made by the CV methods employed in this study, both of which presume that the training data adequately represents the full range of variability in patient conditions. However, when working with data sets that have a limited sample size or lack diversity, this assumption may lead to an incomplete assessment of model performance. In addition, the findings of this study may not be directly generalizable to broader TBI populations, as factors such as injury severity and variations in treatment protocols could influence physiological signal dynamics, introducing further variability.

Another limitation of this study is the exclusion of BlockedCV from interval prediction. Its rigid data splitting necessitated fold-specific tuning while possibly yielding unstable interval estimates. Moreover, adapting the BlockedCV implementation for interval prediction proved technically problematic, frequently generating errors during model fitting and evaluation. Due to the considerable time required to resolve these persistent issues and the minimal improvements in performance and reliability, we decided to exclude this method altogether for interval prediction. This exclusion limits our ability to assess performance of BlockedCV in interval prediction tasks.

In addition, the data augmentation techniques applied to handle missing data points may have impacted the model's performance. Linear interpolation was used for gaps of less than five missing data points, while larger gaps were removed from the data set. This approach assumes linear trends between missing values, which may not always reflect the true physiological dynamics, potentially leading to biases or inaccuracies in the model’s predictions. The impact of this data preprocessing step, especially at lower temporal resolutions, warrants further investigation to assess its effect on the predictive accuracy of ARIMA models.

Moreover, this study did not focus on identifying median optimal ARIMA models with lower *p* and *q* values, which could have provided simpler yet effective forecasting solutions. Similarly, it does not explore the potential impact of external factors, such as patient demographics, treatment interventions, or underlying medical conditions, that may influence the time-series characteristics of cerebral physiological signals. Although these limitations could significantly contribute to model performance, they remain outside the scope of this analysis.

## Future directions

Building on these findings, future research should focus on developing hybrid modeling approaches that integrate ARIMA with advanced machine learning techniques, such as deep learning models, ensemble methods, or state-space frameworks. These hybrid strategies could help overcome ARIMA’s limitations in capturing long-term dependencies and handling the increased variability seen in raw physiological signals at lower temporal resolutions. In addition, incorporating external covariates, such as patient demographics, treatment interventions, or multimodal physiological data, could further refine model predictions and enhance their clinical relevance. Given the non-linearity and abrupt fluctuations observed in raw cerebral physiological signals, exploring non-linear models or hybrid approaches may provide better predictive performance, particularly for signals with irregular patterns. For example, hybrid ARIMA–machine learning models (such as long short-term memory (LSTM)) can pair ARIMA’s strength in capturing linear autocorrelations with LSTM network’s ability to learn nonlinear and time-varying dynamics. Similarly, state-space or ARIMA–Kalman filter frameworks may offer adaptive forecasting capabilities that respond more effectively to sudden physiologic transitions or evolving autoregulatory states. These represent feasible next steps for developing more robust forecasting tools in neurocritical care.

Addressing these challenges is crucial for both improving the robustness and accuracy of time-series forecasting in cerebral physiology and enabling real time, data-driven decision-making in critical care settings. Future studies should also explore machine learning techniques to tailor forecasting models to individual patient trajectories, ultimately leading to more personalized and responsive monitoring strategies for TBI management.

## Conclusion

The findings of this study suggest that ARIMA performs best at high temporal resolutions, particularly for derived CVR indices, with predictive accuracy declining for raw physiological signals as resolution decreases. The choice of CV method further influences forecasting performance, with BlockedCV demonstrating robustness in long-term predictions despite increased variability, and TSS striking a balance between short-term and long-term forecasting effectiveness. However, all methods face challenges at the lowest resolutions, underscoring the limitations of traditional ARIMA models for long-term forecasting of complex physiological signals. In addition, computational efficiency varies with prediction type and CV strategy, with longer training times observed for interval predictions and TSS-based modeling.

## Supplementary Information

Additional file1 (DOCX 1908 KB)

## Data Availability

The data that support the findings of this study are not openly available due to reasons of sensitivity. Requests to access the data sets should be directed to senior author Dr. Frederick A. Zeiler.

## Abbreviations

1stepCV: One-step cross validation
ABP: Arterial blood pressure
ACF: Autocorrelation function
ADF: Augmented Dickey–Fuller
AIC: Akaike information criterion
AMP: Intracranial pressure pulse waveform
ARIMA: Autoregressive integrated moving average
BlockedCV: Blocked time-series cross validation
CAHR–TBI: Canadian high-resolution traumatic brain injury
CBF: Cerebral blood flow
CHREB: University of Calgary Conjoint Health Research Ethics Board
CPP: Cerebral perfusion pressure
CREB: University of British Columbia Clinical Research Ethics Board
CSV: Comma separated value
CV: Cross validation
CVR: Cerebrovascular reactivity
*d*: Differencing order
DFT: Discrete Fourier transform
EVD: External ventricular drain
HREB: University of Manitoba Health Research Ethics Board
ICM +: Intensive Care Monitoring “Plus”
ICP: Intracranial pressure
ICU: Intensive care unit
GCS: Glasgow coma scale
KPSS: Kwiatkowski–Phillips–Schmidt–Shin
MAE: Mean absolute error
MAP: Mean arterial pressure
*p*: Autoregressive order
PACF: Partial autocorrelation function.
PAx: Pulse amplitude index
PbtO_2_: Cerebral oxygen saturation
PRx: Pressure reactivity index
R^2^: R^2^ score
RAC: Cerebral autoregulation index
RAP: Index of cerebral compensatory reserve
RMSE: Root mean square error
TSS: TimeSeriesSplit
*q*: Moving average order
