# Supplementary material for: ARIMA-based forecasting of cerebral physiologic signals in acute traumatic brain injury: a CAnadian high-resolution TBI (CAHR–TBI) cohort study
Source: Intensive Care Med Exp. 2026 Mar 2;14:24. doi: 10.1186/s40635-026-00855-y (PMC12953804; doi:10.1186/s40635-026-00855-y)
Supplement: Supplementary file 1 — Additional file1 (DOCX 1908 KB) [file 40635_2026_855_MOESM1_ESM.docx]

Appendix A

**Acronyms and Abbreviations**

1stepCV: 1-step cross validation

ACF: autocorrelation function

AIC: Akaike information criterion

ARIMA: autoregressive integrated moving average

BlockedCV: blocked time-series cross validation

CPP: cerebral perfusion pressure

ICP: intracranial pressure

MAE: mean absolute error

MAP: mean arterial pressure

PACF: partial autocorrelation function.

PAx: pulse amplitude index

PbtO_2_: cerebral oxygen saturation

PRx: pressure reactivity index

R^2^: R^2^ score

RAC: cerebral autoregulation index

RAP: index of cerebral compensatory reserve

RMSE: root mean square error

TSS: TimeSeriesSplit.


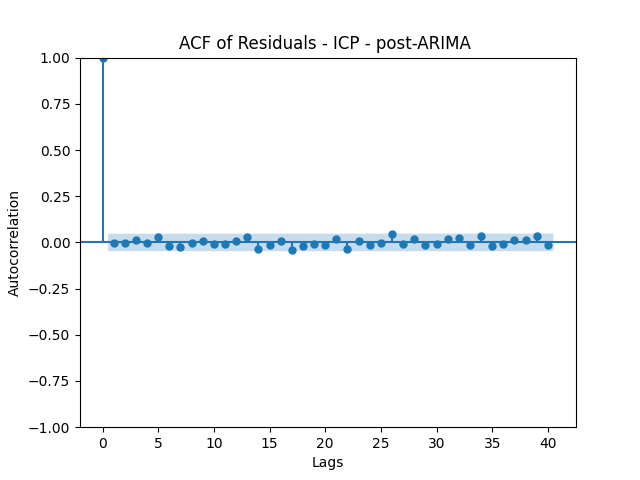

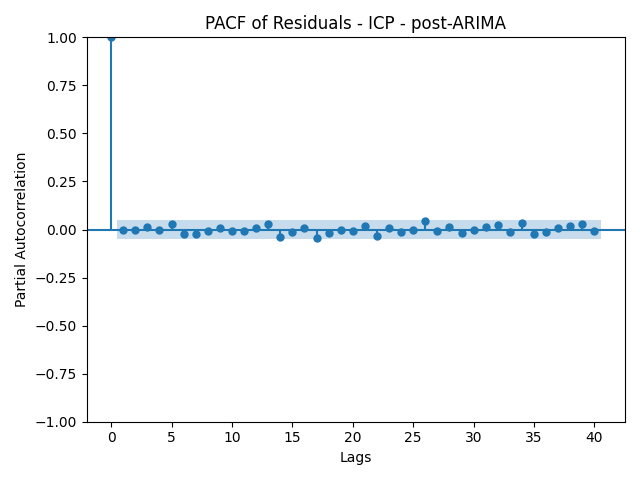

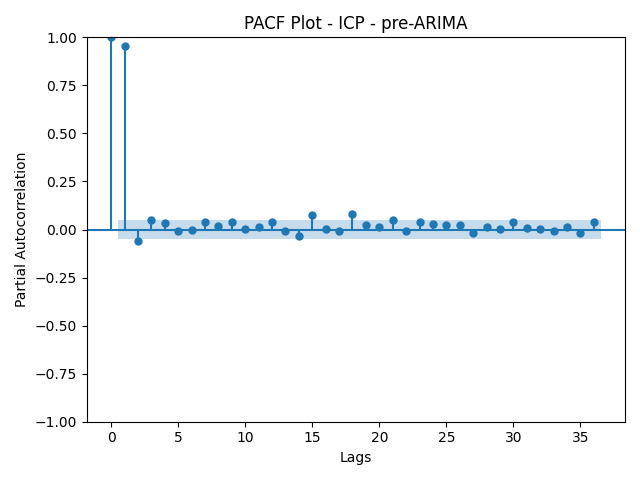

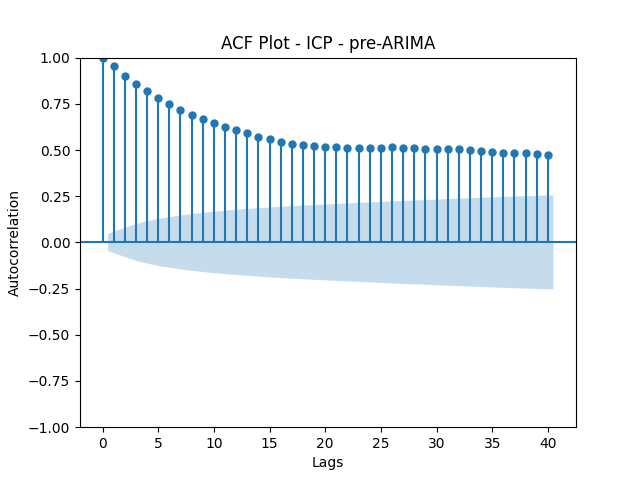


(A)

(B)

(A)

(C)

(B)

(D)

Figure A. 1 -The ACF and PACF plots for the 5-minute point-sampled ICP signal are presented both before and after ARIMA modeling. Panels (A) and (B) display the pre-ARIMA ACF and PACF plots, respectively, while panels (C) and (D) show the corresponding plots following the application of ARIMA with the median optimal parameters: an autoregressive (*p*) order of 5, an integrative (*d*) order of 1, and a moving average (*q*) order of 7. Post-ARIMA, the plots reveal a substantial reduction in significant lags, reflecting an improved model fit.


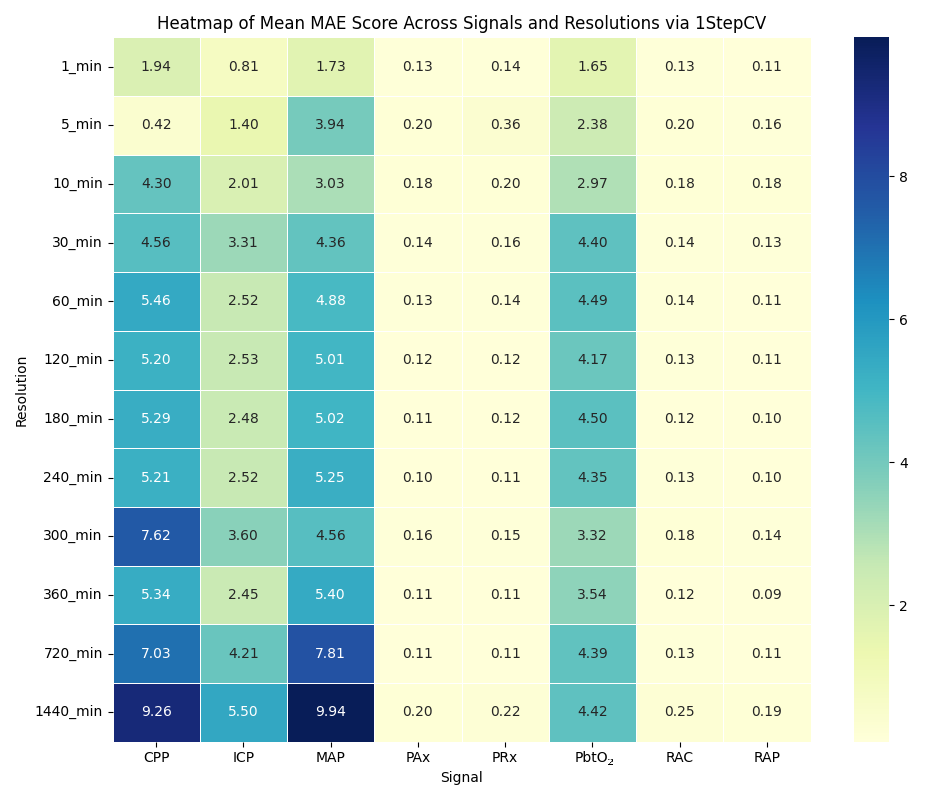


Figure A. 2 - Heatmap of the MAE across different cerebral physiological signals and temporal resolutions using 1stepCV for point prediction


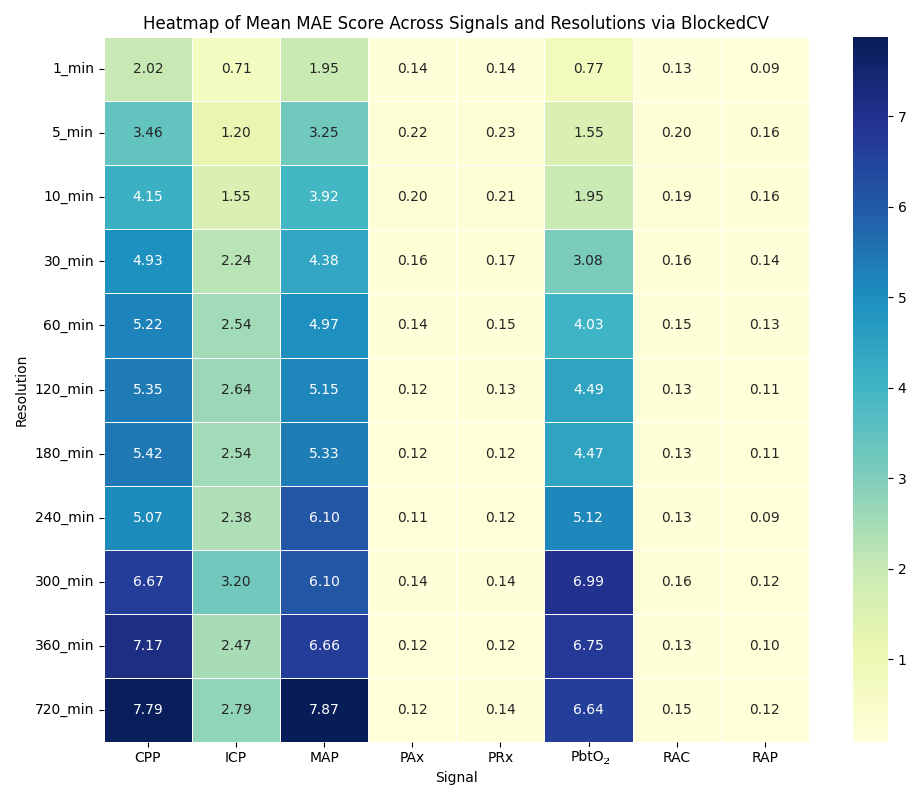


Figure A. 3 - Heatmap of the MAE across different cerebral physiological signals and temporal resolutions using BlockedCV for point prediction


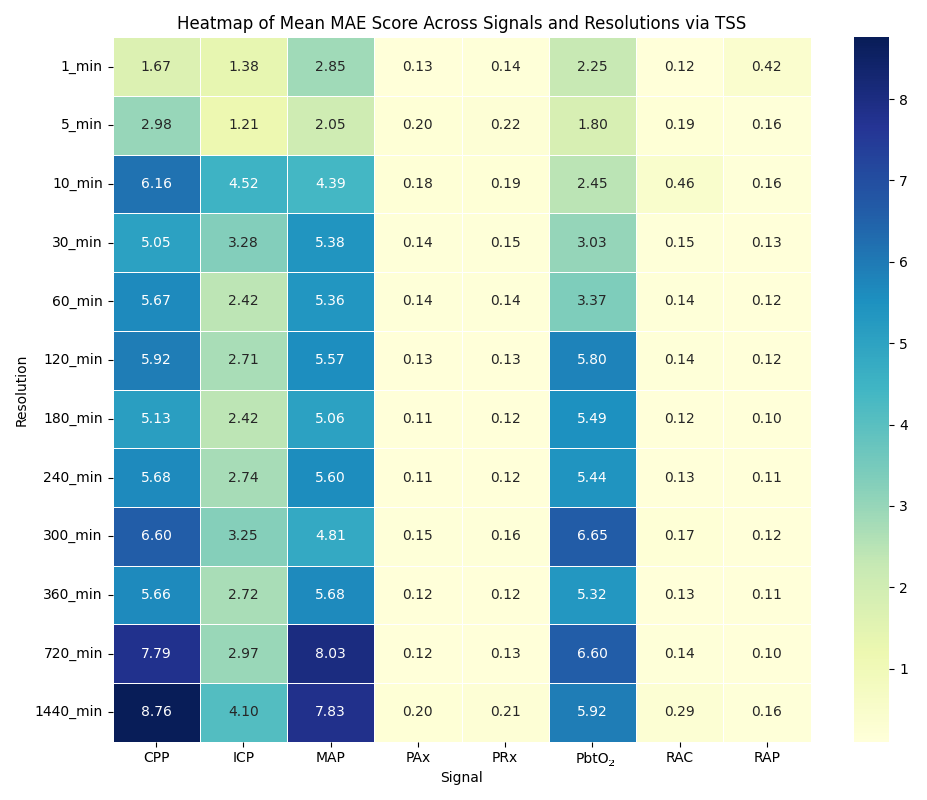


Figure A. 4 - Heatmap of the MAE across different cerebral physiological signals and temporal resolutions using TSS for point prediction


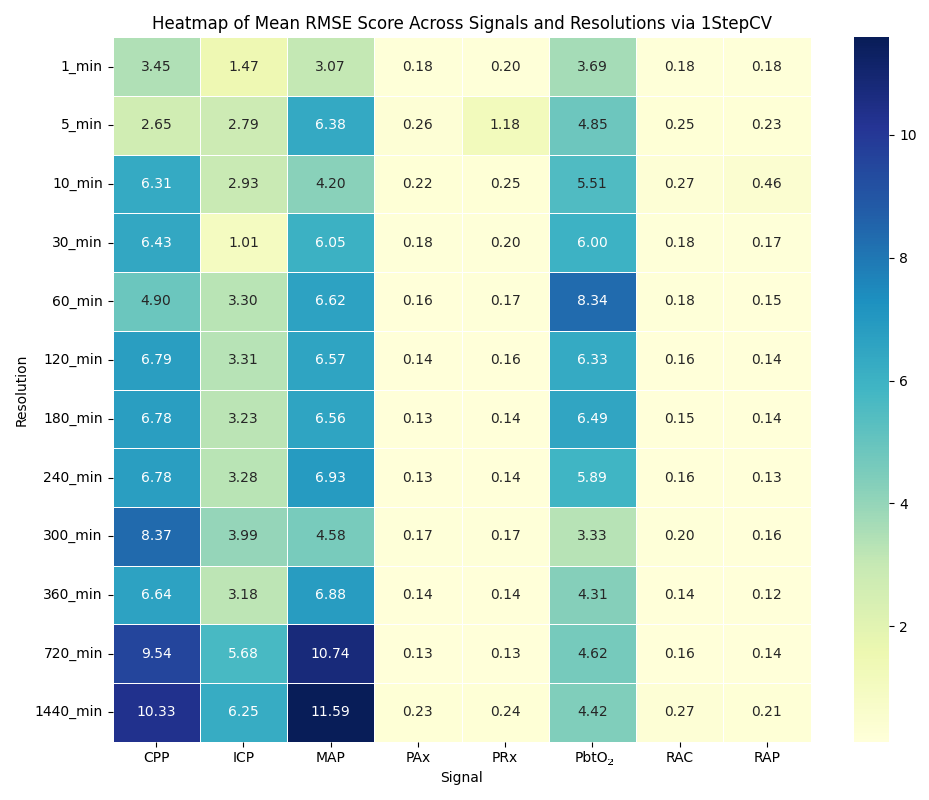


Figure A. 5 - Heatmap of the RMSE across different cerebral physiological signals and temporal resolutions using 1stepCV for point prediction


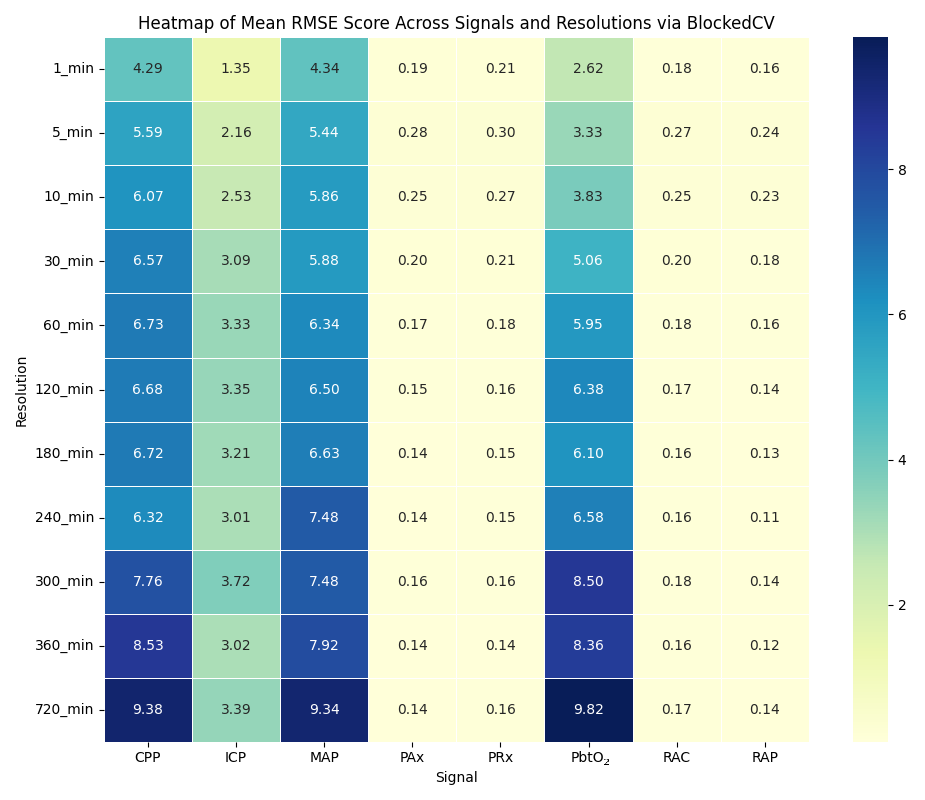


Figure A. 6 - Heatmap of the RMSE across different cerebral physiological signals and temporal resolutions using BlockedCV for point prediction


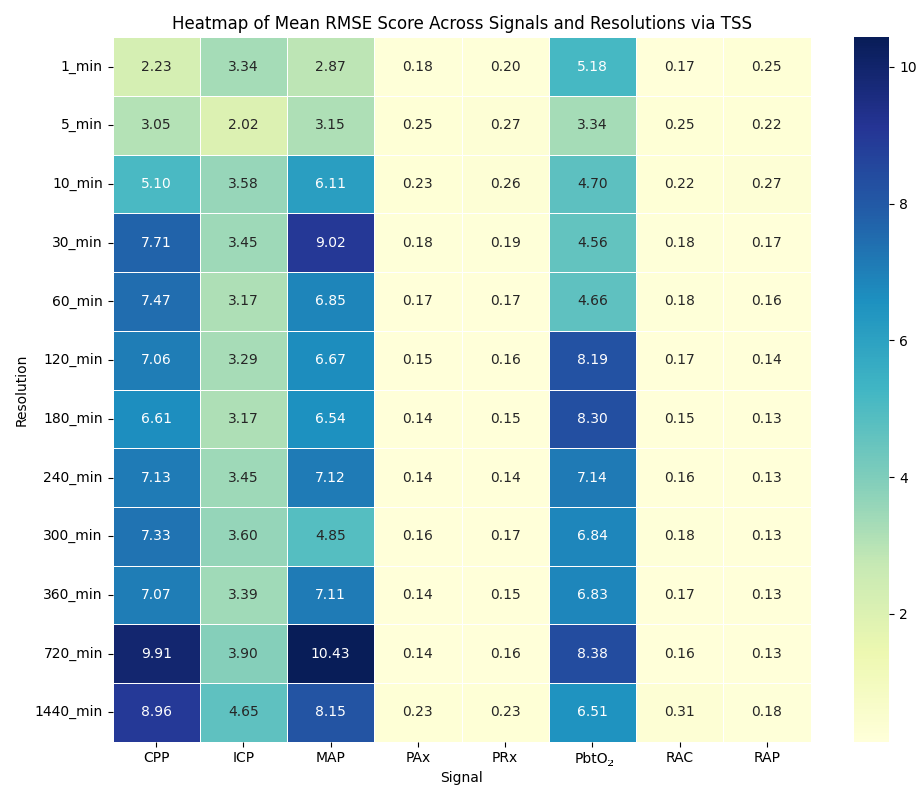


Figure A. 7 - Heatmap of the RMSE across different cerebral physiological signals and temporal resolutions using TSS for point prediction


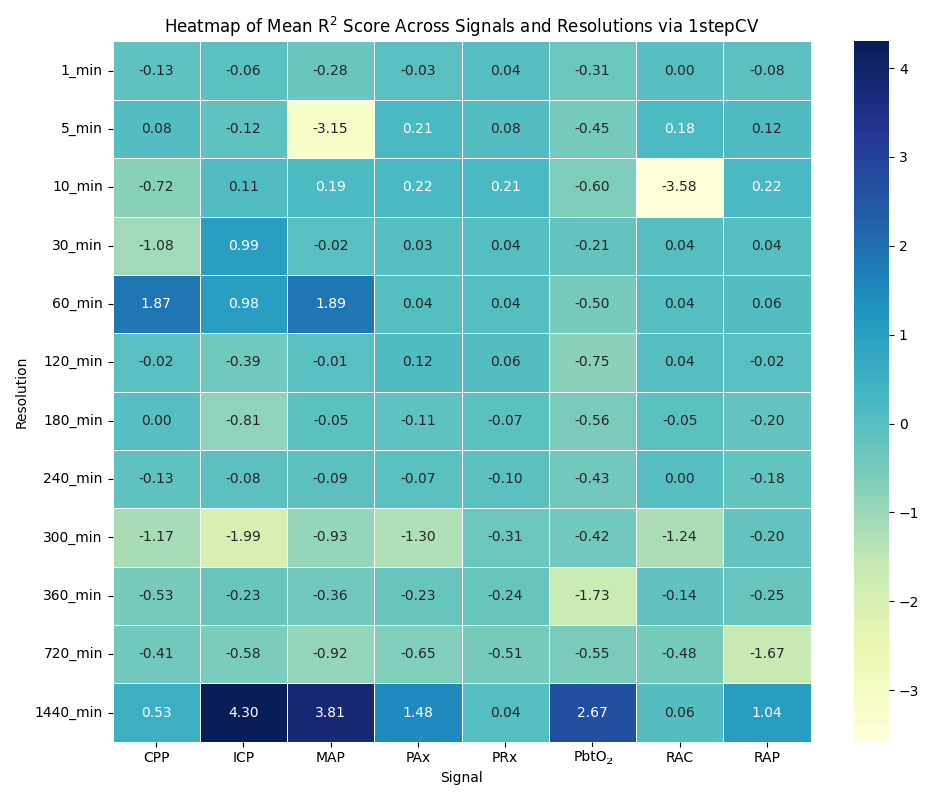


Figure A. 8 - Heatmap of the R^2^ across different cerebral physiological signals and temporal resolutions using 1stepCV for point prediction


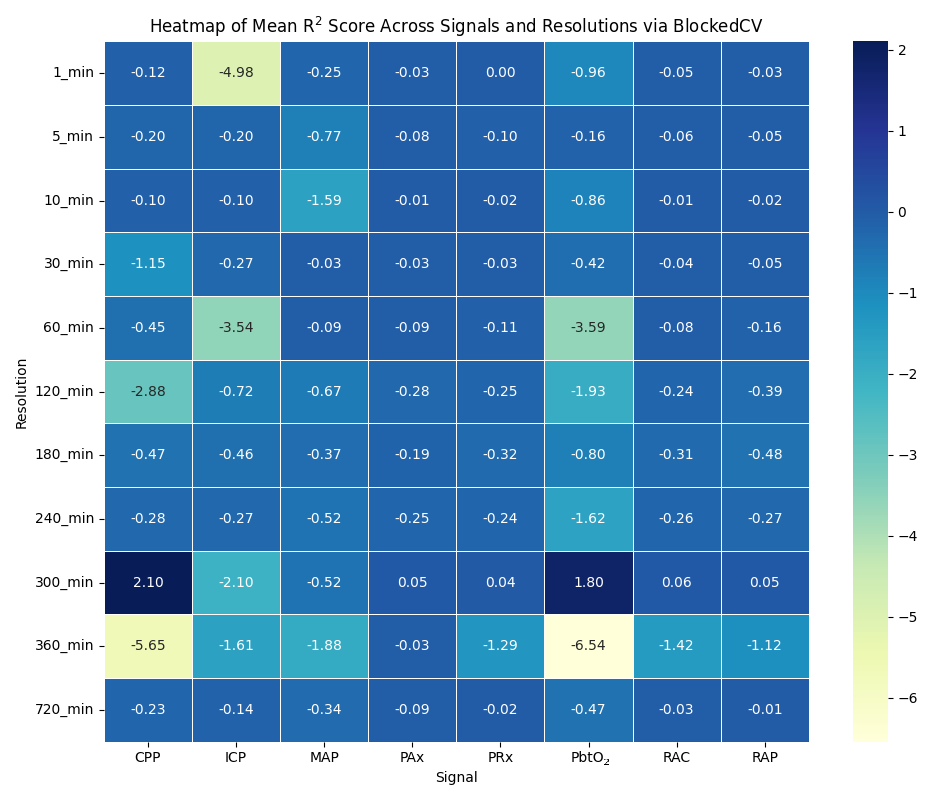


Figure A. 9 - Heatmap of the R^2^ across different cerebral physiological signals and temporal resolutions using BlockedCV for point prediction


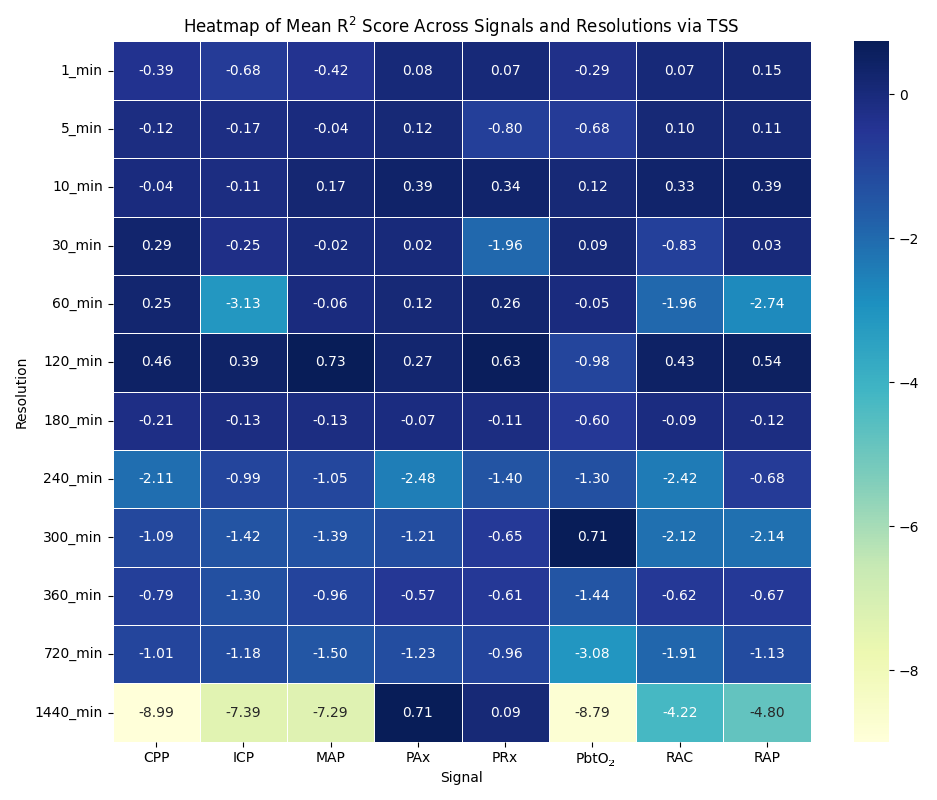


Figure A. 10 - Heatmap of the R^2^ across different cerebral physiological signals and temporal resolutions using TSS for point prediction


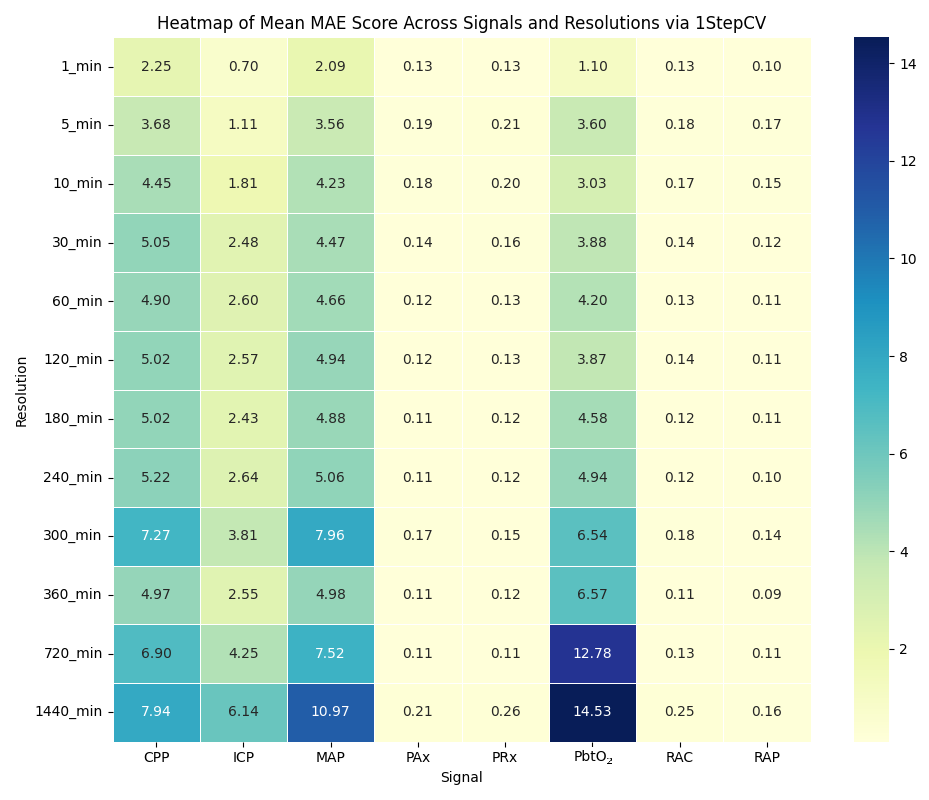


Figure A. 11 - Heatmap of the MAE across different cerebral physiological signals and temporal resolutions using 1stepCV for interval prediction


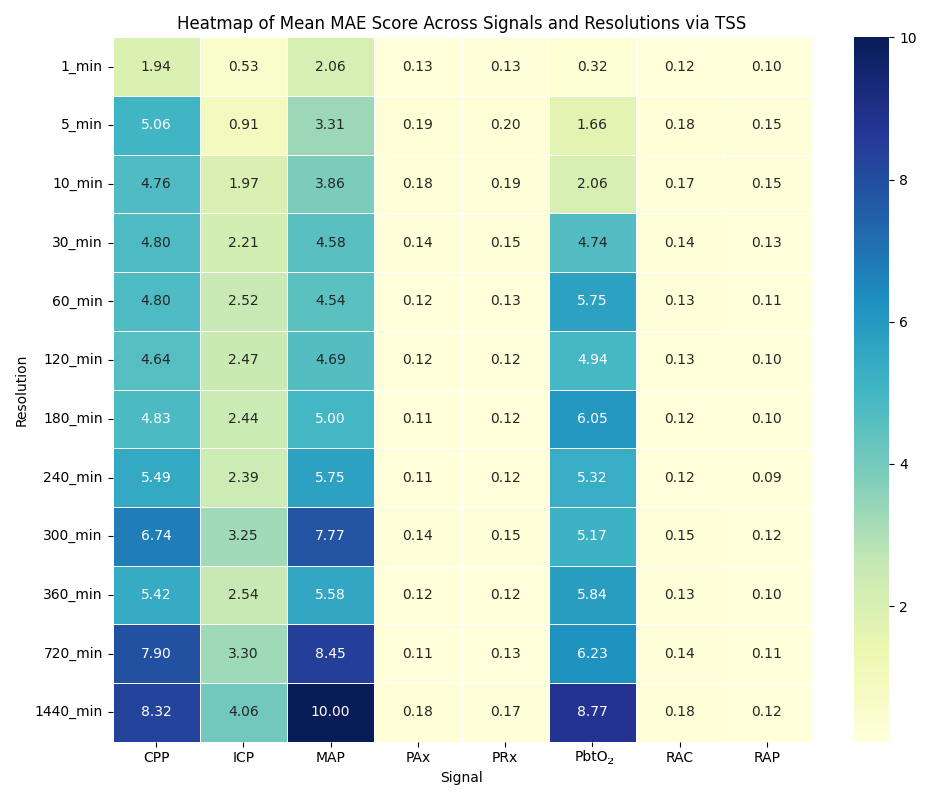


Figure A. 12 - Heatmap of the MAE across different cerebral physiological signals and temporal resolutions using TSS for interval prediction


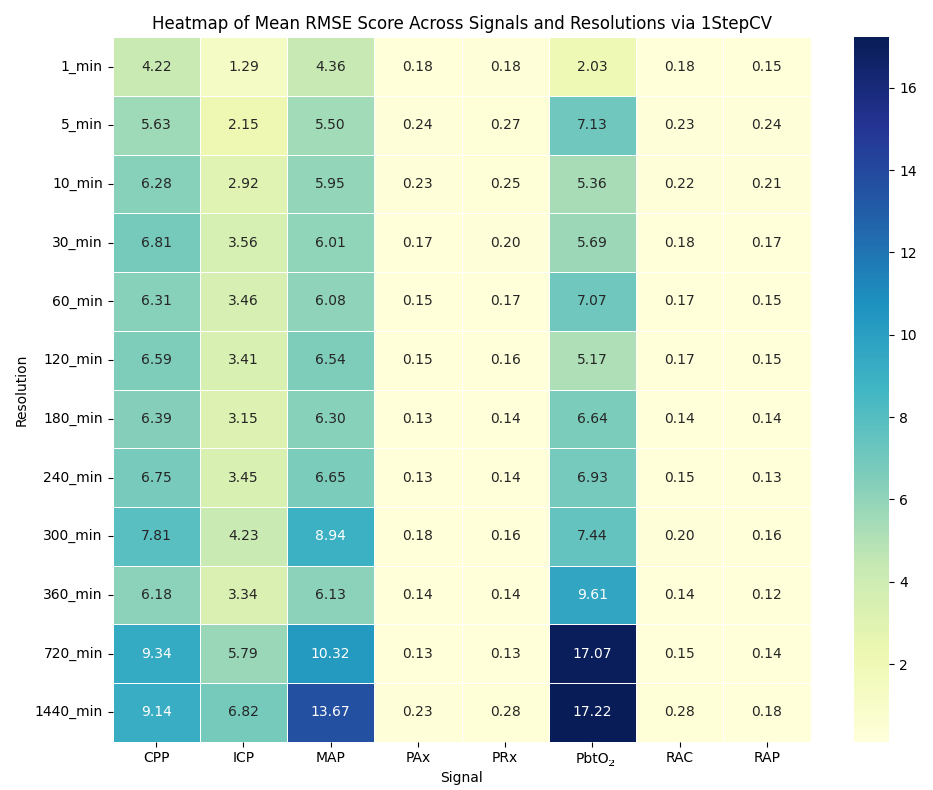


Figure A. 13 - Heatmap of the RMSE across different cerebral physiological signals and temporal resolutions using 1stepCV for interval prediction


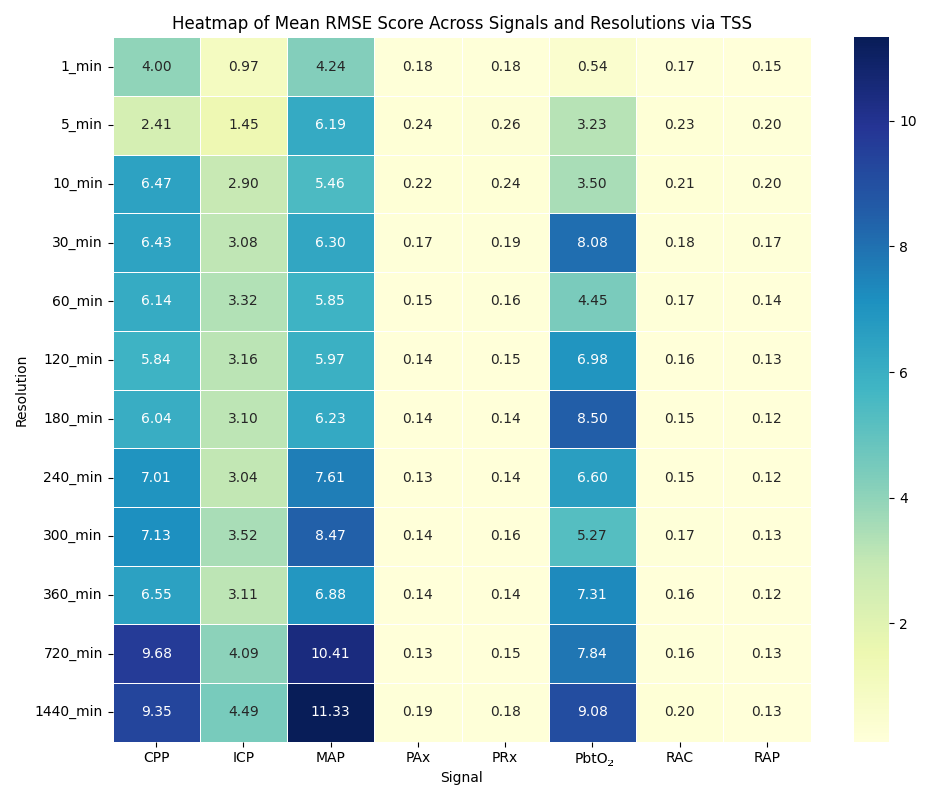


Figure A. 14 - Heatmap of the RMSE across different cerebral physiological signals and temporal resolutions using TSS for interval prediction


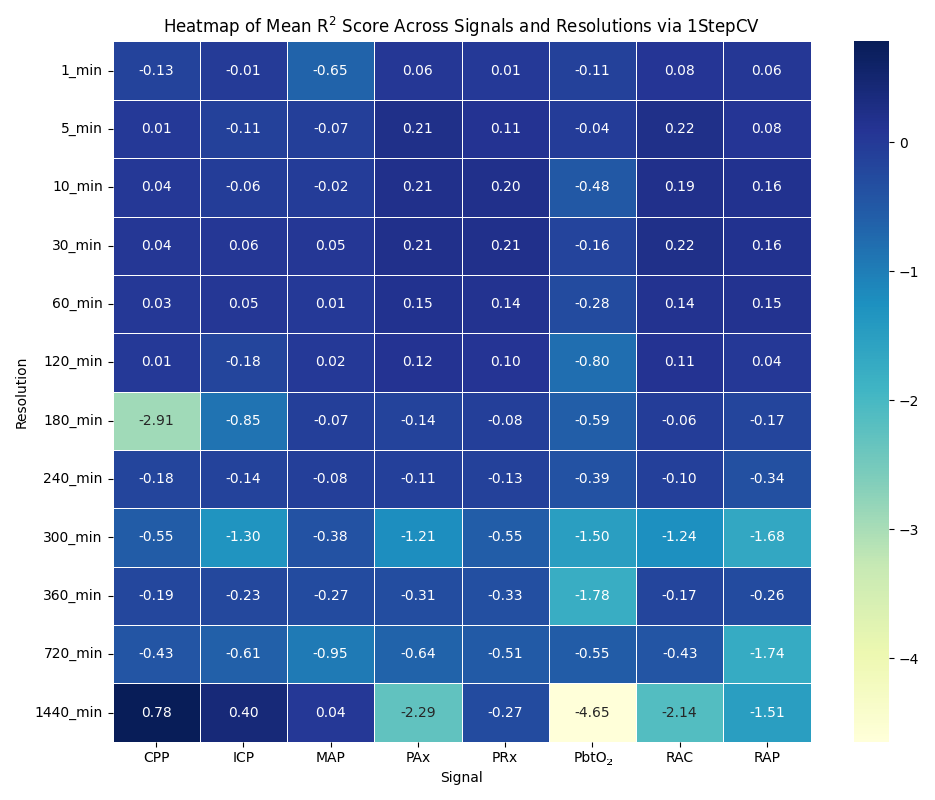


Figure A. 15 - Heatmap of the R^2^ across different cerebral physiological signals and temporal resolutions using 1stepCV for interval prediction


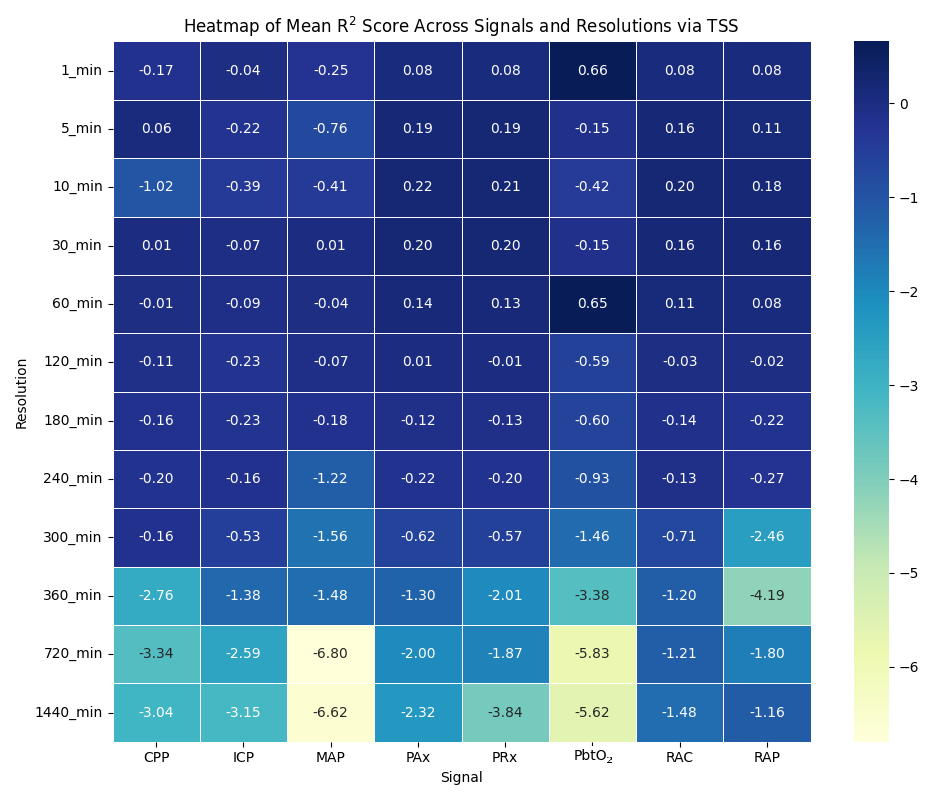


Figure A. 16 - Heatmap of the R^2^ across different cerebral physiological signals and temporal resolutions using TSS for interval prediction


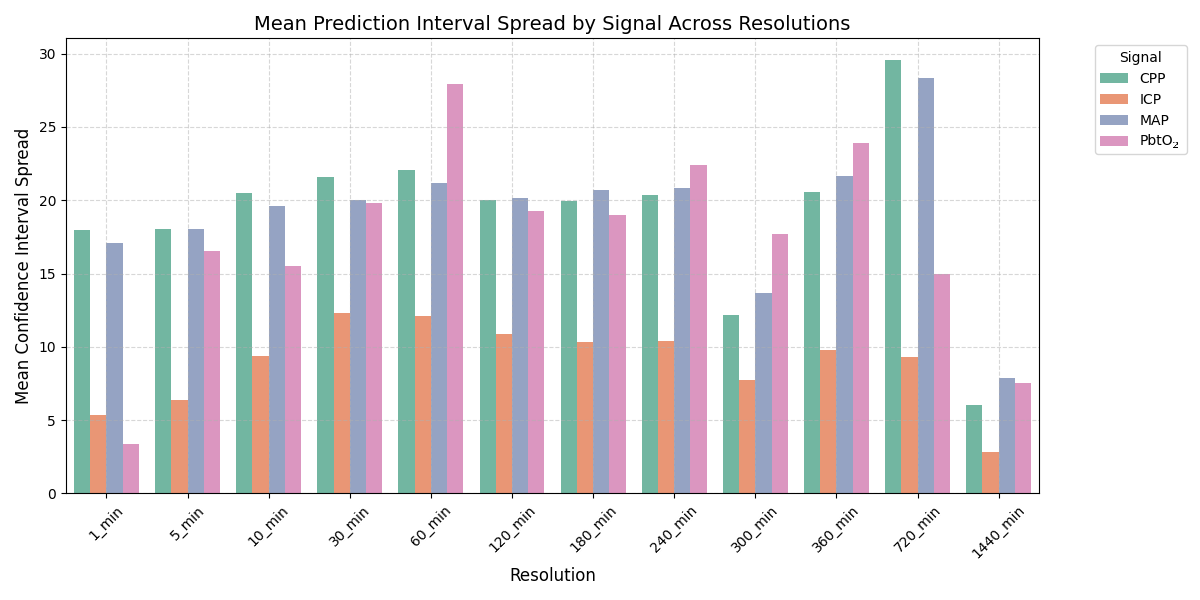


Figure A. 17 - Bar plot of CI of raw signals spread across different temporal resolutions with 1StepCV for interval prediction


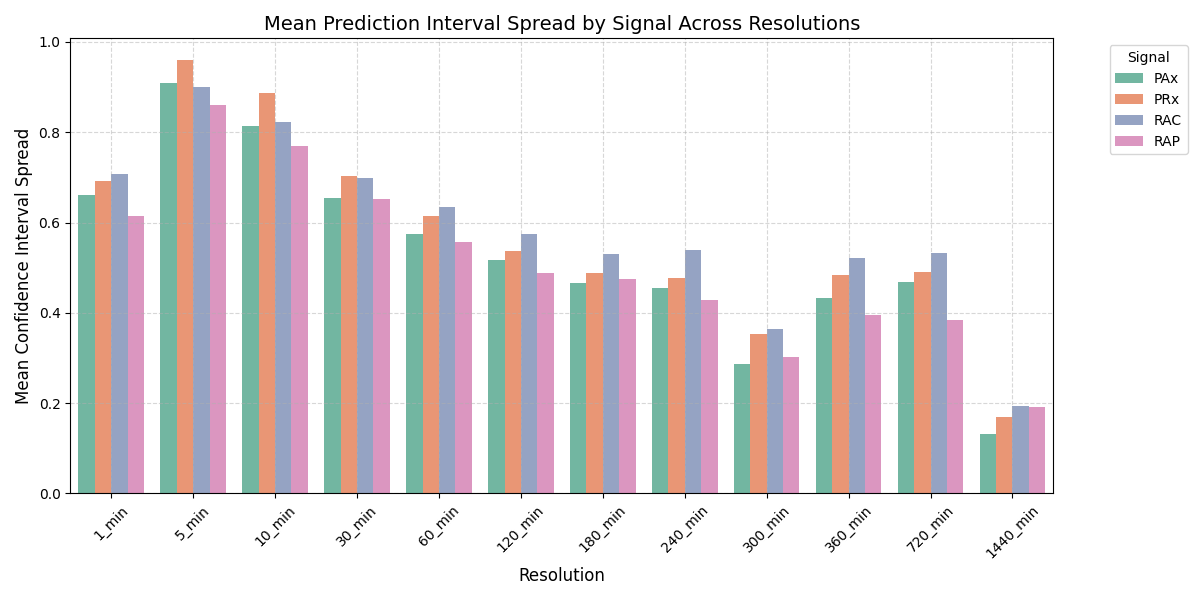


Figure A. 18 - Bar plot of CI of derived signals spread across different temporal resolutions with 1StepCV for interval prediction


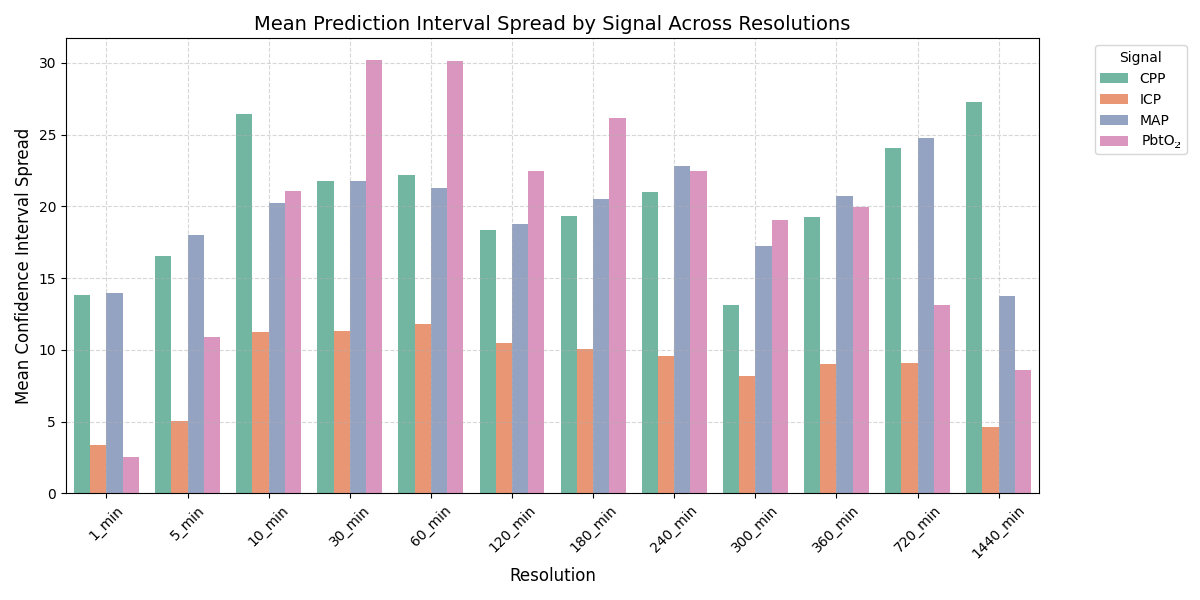


Figure A. 19 - Bar plot of CI of raw signals spread across different temporal resolutions with TSS for interval prediction


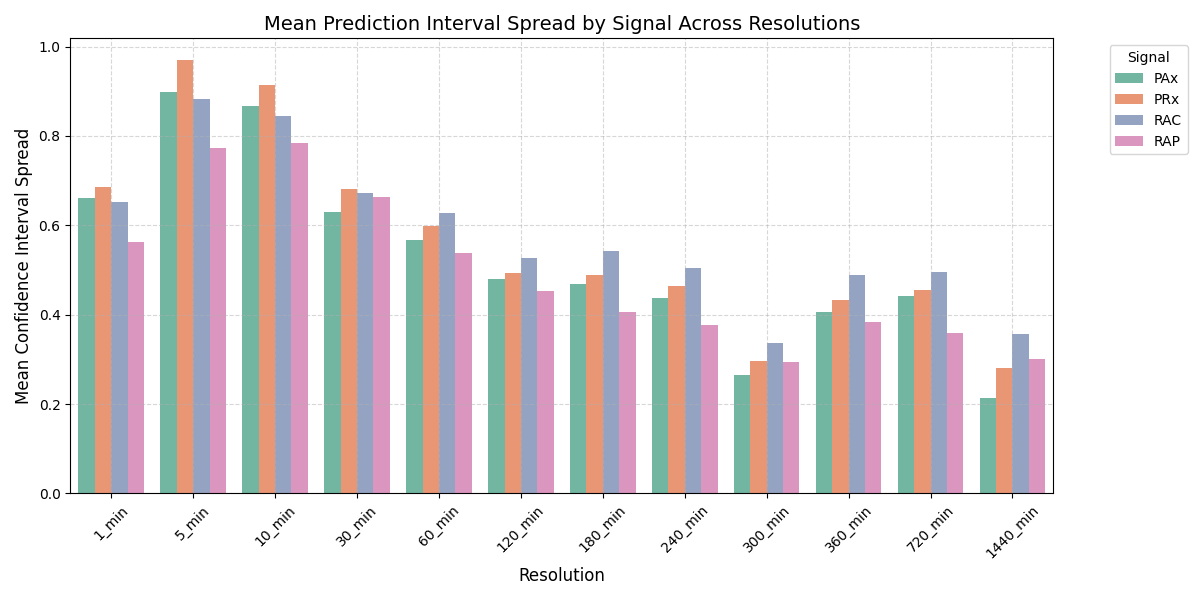


Figure A. 20 - Bar plot of CI of derived signals spread across different temporal resolutions with TSS for interval prediction

Table A. 1 - Median optimal model parameters for all signals across all resolutions, determined using AIC.

| **Signal** | **1-min** | **5-min** | **10-min** | **30-min** | **1-hour** | **2-hour** | **3-hour** | **4-hour** | **5-hour** | **6-hour** | **12-hour** | **1-day** |
| --- | --- | --- | --- | --- | --- | --- | --- | --- | --- | --- | --- | --- |
| MAP | (4,1,5)\|32437.6512 | (4,1,4)\|7566.1234 | (3,1,4)\|3771.6399 | (3,1,4)\|1340.8527 | (3,1,3)\|689.3287 | (3,1,2)\|359.4441 | (4,1,2)\|234.2886 | (4,2,2)\|175.9431 | (4,2,2)\|145.8193 | (4,2,2)\|113.2266 | (5,2,2)\|53.1833 | (4,1,1)\|21.1179 |
| ICP | (5,1,6)\|19465.3870 | (4,1,4)\|5151.5914 | (3,1,4)\|2715.2870 | (3,1,3)\|972.4728 | (3,1,3)\|517.8965 | (3,1,2)\|260.7877 | (3,1,2)\|175.4765 | (4,1,2)\|131.0412 | (4,1,1)\|109.9212 | (4,1,1)\|85.5492 | (5,1,1)\|38.9008 | (4,1,1)\|15.3208 |
| CPP | (4,1,5)\|31589.4163 | (4,1,4)\|7020.9794 | (3,1,4)\|3614.7909 | (3,1,3)\|1295.3518 | (3,1,3)\|637.6638 | (3,1,2)\|331.0755 | (4,1,2)\|220.7812 | (4,2,2)\|162.8390 | (4,2,2)\|136.4757 | (4,2,2)\|107.2640 | (5,2,2)\|48.0941 | (4,1,1)\|18.5961 |
| PRx | (5,1,3)\|-2705.5415 | (3,1,3)\|214.4056 | (2,1,2)\|-28.8594 | (2,1,2)\|-111.1637 | (2,1,2)\|-83.3895 | (3,1,2)\|-55.5690 | (3,1,1)\|-40.2198 | (3,1,1)\|-32.0176 | (3,1,1)\|-28.0848 | (3,1,1)\|-23.6506 | (4,1,1)\|-13.6186 | (3,1,1)\|-10.1447 |
| PAx | (5,1,3)\|-3317.5719 | (3,1,2)\|51.3212 | (2,1,2)\|-116.8506 | (2,1,2)\|-136.3830 | (2,1,2)\|-94.4192 | (2,1,2)\|-60.2984 | (3,1,1)\|-43.6922 | (3,1,1)\|-35.0696 | (3,1,1)\|-29.8718 | (3,1,1)\|-25.3364 | (4,1,1)\|-14.9201 | (3,1,1)\|-11.7097 |
| RAC | (5,1,4)\|-4173.7097 | (3,1,3)\|-45.2354 | (2,1,2)\|-132.4741 | (2,1,2)\|-120.6020 | (2,1,2)\|-80.1245 | (3,1,1)\|-49.4206 | (3,1,1)\|-35.5625 | (3,1,1)\|-27.7896 | (3,1,1)\|-24.4844 | (3,1,1)\|-20.5554 | (4,1,1)\|-12.2832 | (3,1,1)\|-10.0164 |
| RAP | (5,1,4)\|-6564.4567 | (3,1,3)\|-408.7564 | (2,1,2)\|-277.6390 | (2,1,2)\|-169.5420 | (2,1,2)\|-111.5923 | (2,1,1)\|-66.3179 | (2,1,1)\|-48.1936 | (3,1,1)\|-37.3456 | (3,1,1)\|-32.6441 | (3,1,1)\|-26.6239 | (3,1,1)\|-15.1382 | (3,1,0)\|-11.0359 |
| PbtO_2_ | (4,1,5)\|22868.8849 | (4,1,4)\|5600.2201 | (3,1,4)\|2845.0132 | (3,1,3)\|1109.2239 | (3,1,3)\|569.8618 | (3,1,2)\|293.0777 | (3,1,2)\|204.1008 | (3,2,2)\|142.8140 | (3,2,1)\|126.6797 | (4,2,2)\|96.4337 | (5,2,1)\|42.9938 | (4,1,1)\|16.8122 |

Table A. 2 - Statistical comparison of ARIMA prediction errors (MAE) across patient subgroups defined by IMPACT variables

| **1saCV results** | | | | | | | | |
| --- | --- | --- | --- | --- | --- | --- | --- | --- |
|  | T_statistic | p-value | T_statistic | p-value | T_statistic | p-value | F_statistic | p-value |
| Resolution | Marshall CT score (group 1: <5, group 2: >=5) | | Sex (group 1: Male, group 2: Female) | | Age (group 1: >=40, group 2: <40) | | ANOVA (Group 1: Bilat Reactive, Group 2: Bilat Unreactive, Group 3: Unilateral Unreactive) | |
| 1_min | 1.44 | 0.15 | -0.4 | 0.69 | -0.04 | 0.97 | 1.34 | 0.26 |
| 5_min | 1 | 0.32 | -1 | 0.32 | -1 | 0.32 | 2.62 | 0.07 |
| 10_min | 1.79 | 0.07 | 1.76 | 0.08 | 0.64 | 0.52 | 0.31 | 0.74 |
| 30_min | 1.79 | 0.07 | 0.19 | 0.85 | -1.84 | 0.07 | 1.09 | 0.34 |
| 60_min | 1.13 | 0.26 | 1.31 | 0.19 | -1.15 | 0.25 | 0.22 | 0.8 |
| 120_min | 0.31 | 0.76 | 1.22 | 0.22 | -0.27 | 0.79 | 0.48 | 0.62 |
| 180_min | 1.4 | 0.16 | 1.49 | 0.14 | -0.56 | 0.58 | 0.02 | 0.98 |
| 240_min | -0.48 | 0.63 | -0.2 | 0.84 | -0.57 | 0.57 | 0.12 | 0.88 |
| 300_min | -0.58 | 0.56 | -1.18 | 0.24 | -0.3 | 0.76 | 0.65 | 0.52 |
| 360_min | -0.04 | 0.97 | 0.1 | 0.92 | -0.54 | 0.59 | 0.62 | 0.54 |
| 720_min | 0.72 | 0.47 | -1.84 | 0.07 | -1.22 | 0.22 | 1.93 | 0.17 |
| 1440_min | 1.75 | 0.08 | 0.04 | 0.97 | -0.19 | 0.85 | 2.48 | 0.07 |
| **BlockedCV results** | | | | | | | | |
|  | T_statistic | p-value | T_statistic | p-value | T_statistic | p-value | F_statistic | p-value |
| Resolution | Marshall CT score (group 1: <5, group 2: >=5) | | Sex (group 1: Male, group 2: Female) | | Age (group 1: >=40, group 2: <40) | | ANOVA (Group 1: Bilat Reactive, Group 2: Bilat Unreactive, Group 3: Unilateral Unreactive) | |
| 1_min | 1.06 | 0.29 | 0.95 | 0.34 | 1.15 | 0.25 | 0.45 | 0.64 |
| 5_min | -1.06 | 0.29 | -1.14 | 0.25 | 1.06 | 0.29 | 1.09 | 0.34 |
| 10_min | 1.47 | 0.14 | 1.08 | 0.28 | 0.37 | 0.71 | 0.62 | 0.54 |
| 30_min | 1.21 | 0.23 | -1.12 | 0.26 | -1.64 | 0.1 | 0.74 | 0.48 |
| 60_min | 1.47 | 0.14 | 1.04 | 0.3 | -0.19 | 0.85 | 1.88 | 0.15 |
| 120_min | -1.29 | 0.2 | 1.21 | 0.23 | 0.37 | 0.71 | 2.69 | 0.07 |
| 180_min | -1.3 | 0.19 | 1.87 | 0.06 | 0.47 | 0.64 | 0.06 | 0.94 |
| 240_min | -0.16 | 0.87 | -0.6 | 0.55 | 1.47 | 0.14 | 1.65 | 0.19 |
| 300_min | 1.2 | 0.23 | 1.21 | 0.23 | -0.46 | 0.64 | 1.18 | 0.30 |
| 360_min | -1.12 | 0.26 | 1.15 | 0.25 | 1.21 | 0.23 | 2.70 | 0.07 |
| 720_min | 0.55 | 0.59 | 1.87 | 0.06 | 0.12 | 0.9 | 2.69 | 0.10 |
| **TSS results** | | | | | | | | |
|  | T_statistic | p-value | T_statistic | p-value | T_statistic | p-value | F_statistic | p-value |
| Resolution | Marshall CT score (group 1: <5, group 2: >=5) | | Sex (group 1: Male, group 2: Female) | | Age (group 1: >=40, group 2: <40) | | ANOVA (Group 1: Bilat Reactive, Group 2: Bilat Unreactive, Group 3: Unilateral Unreactive) | |
| 1_min | 1.27 | 0.21 | 1.27 | 0.21 | -1.27 | 0.21 | 0.45 | 0.64 |
| 5_min | 1.27 | 0.21 | 1.27 | 0.21 | -1.27 | 0.21 | 0.50 | 0.61 |
| 10_min | 1.19 | 0.23 | -1.17 | 0.24 | -1.15 | 0.25 | 0.39 | 0.68 |
| 30_min | 0.47 | 0.64 | -1.49 | 0.14 | -1.15 | 0.25 | 0.06 | 0.94 |
| 60_min | 1.48 | 0.14 | 0.37 | 0.71 | -1.63 | 0.1 | 2.70 | 0.07 |
| 120_min | -1.61 | 0.11 | 0.58 | 0.56 | -1.78 | 0.08 | 1.25 | 0.19 |
| 180_min | 0.37 | 0.71 | -0.52 | 0.61 | -0.32 | 0.75 | 0.92 | 0.40 |
| 240_min | 0.47 | 0.64 | 0.45 | 0.65 | 1.48 | 0.14 | 2.41 | 0.07 |
| 300_min | -1.15 | 0.25 | 1.87 | 0.06 | -0.46 | 0.65 | 2.69 | 0.07 |
| 360_min | -1.73 | 0.08 | -0.46 | 0.65 | 1.08 | 0.28 | 1.20 | 0.30 |
| 720_min | 0.93 | 0.36 | 1.08 | 0.28 | 1.13 | 0.26 | 3.79 | 0.08 |
| 1440_min | 1.46 | 0.15 | 0.77 | 0.44 | -1.42 | 0.16 | 0.60 | 0.44 |

Table A. 3 - Median computational times for point and internal predictions, averaged across patients, for a 1-min temporal resolution.

| Predictions | 1stepCV - Point | | BlockedCV - Point | | TSS - Point | | 1stepCV - Interval | | TSS - Interval | |
| --- | --- | --- | --- | --- | --- | --- | --- | --- | --- | --- |
| Singal | Training (s) | Testing (s) | Training (s) | Testing (s) | Training (s) | Testing (s) | Training (s) | Testing (s) | Training (s) | Testing (s) |
| CPP | 57.21 | 0.16 | 0.98 | 0.01 | 1091.68 | 0.66 | 37.16 | 0.26 | 195.45 | 0.98 |
| ICP | 114.91 | 0.17 | 1.07 | 0.01 | 1534.32 | 0.72 | 70.10 | 0.31 | 391.51 | 1.08 |
| MAP | 100.4 | 0.18 | 1.16 | 0.01 | 4990.01 | 0.75 | 396.96 | 1.20 | 327.18 | 1.06 |
| PAx | 57.11 | 0.16 | 0.66 | 0.01 | 1044.67 | 0.75 | 193.07 | 0.42 | 115.33 | 0.89 |
| PRx | 51.15 | 0.16 | 0.67 | 0.01 | 1090.10 | 0.73 | 68.30 | 0.37 | 50.92 | 0.51 |
| PbtO_2_ | 111.95 | 0.18 | 0.96 | 0.01 | 793.24 | 0.47 | 1389.65 | 1.74 | 565.98 | 1.48 |
| RAC | 52.16 | 0.16 | 0.69 | 0.01 | 1056.54 | 0.74 | 183.12 | 0.49 | 62.59 | 0.61 |
| RAP | 76.79 | 0.18 | 0.95 | 0.01 | 985.50 | 0.77 | 129.72 | 0.48 | 121.67 | 0.80 |
